# Targeting eRNA‐Producing Super‐Enhancers Regulates TNFα Expression and Mitigates Chronic Inflammation in Mice and Patient‐Derived Immune Cells

**DOI:** 10.1002/advs.202505214

**Published:** 2025-07-21

**Authors:** Minjeong Cho, Su Min Kim, Jiyeon Lee, Oh Chan Kwon, Wonjin Woo, Eunji Lee, Hyo Jin Park, Yeongun Lee, So Hee Dho, Tae‐Kyung Kim, Min‐Chan Park, Richard A. Flavell, Lark Kyun Kim

**Affiliations:** ^1^ Department of Biomedical Sciences Graduate School of Medical Science Brain Korea 21 Project Gangnam Severance Hospital Yonsei University College of Medicine Seoul 03722 Republic of Korea; ^2^ Division of Rheumatology Department of Internal Medicine Yonsei University College of Medicine Seoul 03722 Republic of Korea; ^3^ Department of Life Sciences Pohang University of Science and Technology (POSTECH) Pohang Gyeongbuk 37673 Republic of Korea; ^4^ Department of Immunobiology Yale School of Medicine New Haven CT 06520 USA; ^5^ Howard Hughes Medical Institute Yale School of Medicine New Haven CT 06520 USA

**Keywords:** antisense oligonucleotide, chronic inflammation, enhancer RNA, rheumatoid arthritis, tumor necrosis factor alpha

## Abstract

Chronic inflammatory diseases are driven by immune cell dysregulation and overproduction of pro‐inflammatory molecules, such as tumor necrosis factor alpha (TNFα). Super‐enhancers (SEs) and their enhancer RNAs (eRNAs) are critical gene expression regulators and offer therapeutic potential beyond protein‐targeting approaches. This work hypothesizes that targeting eRNAs could reduce chronic inflammation by modulating TNFα expression. This work generates TNF‐9 knockout (KO) mice by deleting a Tnfα‐regulating enhancer region. These mice exhibit significantly reduced Tnfα levels, improved disease outcomes, and diminished immune cell activation in models of rheumatoid arthritis (RA), psoriasis, and lipopolysaccharide (LPS)‐induced sepsis. Integrative epigenomic and transcriptomic analysis identify additional LPS‐responsive, eRNA‐producing enhancers as therapeutic targets. Antisense oligonucleotide (ASO)‐mediated knockdown of TNF‐9 eRNA in mouse macrophages demonstrate decreased Tnfα expression and alleviated RA symptoms. Furthermore, ASO‐mediated inhibition of the eRNA of the human homolog of TNF‐9 similarly reduce TNFα levels. These findings support eRNA‐targeted interventions as potential treatment for chronic inflammatory diseases.

## Graphical abstract

Targeting eRNAs from TNFα‐associated super‐enhancers with ASOs effectively reduces TNFα expression and inflammatory responses in both mouse models and patient‐derived immune cells. The conserved nature of these regulatory regions between species highlights the potential of eRNA‐targeted approaches as a therapeutic strategy for chronic inflammatory diseases

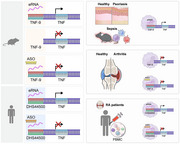

## 1 Introduction

Epigenetic regulation is essential for orchestrating gene expression and influences a wide array of biological processes throughout the lifespan of an organism.^[^ ^1^, ^2^ ^]^ Among its various components, enhancers fine‐tune gene transcription in a cell type‐ and context‐dependent manner.^[^ ^3^, ^4^, ^5^ ^]^ Unlike promoters, which are typically located immediately upstream of target genes, enhancers can reside at considerable distances yet modulate transcription by recruiting transcription factors (TFs) and stabilizing interactions with core transcriptional machinery.^[^ ^6^ ^]^ Through these interactions, enhancers coordinate spatiotemporal gene expression and integrate multiple regulatory cues that govern cell fate, development, and disease progression.

Within this regulatory landscape, super‐enhancers (SEs) have emerged as potent drivers of transcription. SEs comprise clusters of enhancers that act synergistically to drive high‐level expression of genes critical for maintaining cell identity and function.^[^ ^7^, ^8^ ^]^ Their enrichment at key regulatory loci underscores their importance in both normal cellular processes and disease states, where dysregulation can lead to significant pathophysiological consequences.^[^ ^9^ ^]^ However, the vast number of enhancers and the complexity introduced by SEs, combined with their context‐dependent activity, pose major challenges in accurately mapping enhancer–gene relationships.^[^ ^10^ ^]^ Extensive research is required to elucidate these complex regulatory networks and harness their therapeutic potential. In this context, enhancer RNAs (eRNAs), a class of non‐coding RNAs transcribed from active enhancers, have emerged as key regulators of gene expression.^[^ ^11^, ^12^, ^13^ ^]^ By promoting enhancer–promoter looping, recruiting transcriptional machinery, and modulating transcriptional output, eRNAs play a crucial role in both physiological and pathological processes.^[^ ^14^, ^15^, ^16^ ^]^

Traditional drug discovery has primarily focused on well‐characterized protein targets, such as G‐protein‐coupled receptors (GPCRs), enzymes, and protein–protein interactions (PPIs).^[^ ^17^, ^18^, ^19^ ^]^ While small‐molecule therapeutics have been developed for these targets, substantial challenges remain, including structural complexity, off‐target effects, limited selectivity, and bioavailability.^[^ ^20^, ^21^ ^]^ Recent advances in structural biology, genomics, and RNA research have shifted the focus toward targeting previously undruggable molecules, such as TFs and RNA‐based elements.^[^ ^22^ ^]^ TFs are major drug targets owing to their central role in gene regulation; however, their intrinsically disordered domains and lack of well‐defined binding pockets for small molecules complicate small‐molecule design. Moreover, their nuclear localization adds further complexity to efficient drug delivery.^[^ ^23^ ^]^ Therefore, alternative strategies are needed to modulate transcription via enhancer‐associated mechanisms.

Growing evidence supports eRNAs as key regulators of transcription, highlighting their potential as therapeutic targets.^[^ ^24^ ^]^ Unlike the static, sequence‐dependent nature of enhancers or the structurally disordered regions of TFs, eRNAs function as dynamic molecules that can be directly modulated using RNA‐targeting strategies. For instance, antisense oligonucleotides (ASOs) provide a precise method to inhibit eRNA activity while potentially minimizing off‐target effects.^[^ ^25^ ^]^ ASO‐based therapies have demonstrated clinical success in treating genetic disorders, such as spinal muscular atrophy and hereditary transthyretin amyloidosis.^[^ ^26^, ^27^, ^28^ ^]^ However, their application in chronic inflammatory diseases remains largely unexplored. Given that persistent transcriptional dysregulation drives chronic inflammation, targeting eRNAs presents a unique opportunity to regulate the expression of pro‐inflammatory mediators.

Here, we propose eRNAs as a novel therapeutic avenue for modulating transcriptional dysregulation in chronic inflammatory diseases. By directly regulating key disease‐associated genes, eRNAs may overcome the limitations of traditional drug targets in conditions, such as rheumatoid arthritis (RA), psoriasis, and other inflammation‐driven disorders. Furthermore, their potential as biomarkers for diagnosis, patient stratification, and personalized medicine underscores the broader significance of eRNA research in improving clinical outcomes.

## 2 Results

### 2.1 Identification of LPS‐Responsive eRNA‐Producing SEs in Mouse Macrophages

**Figure 1:**
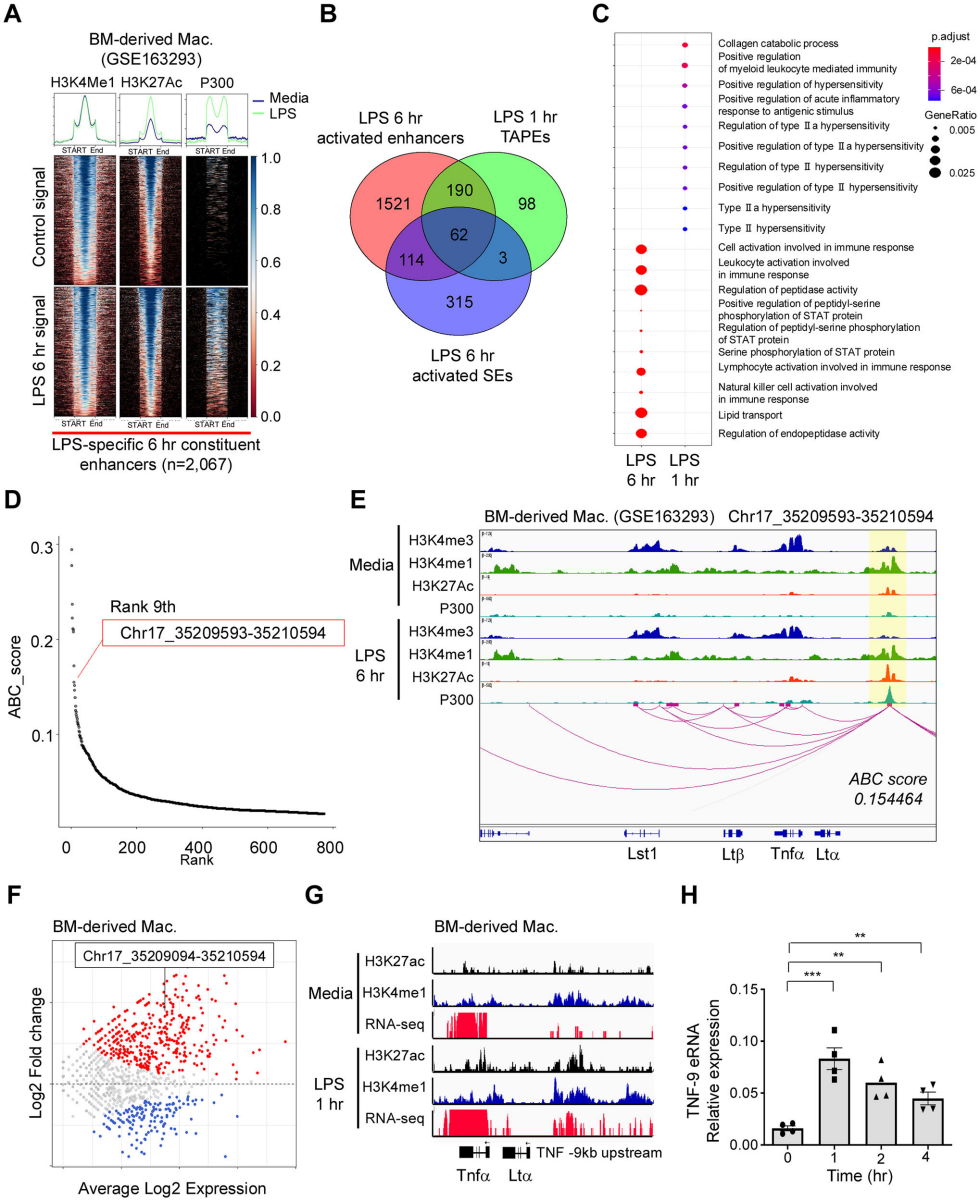
Characterization of the *Tnfα* SE and eRNA in Response to LPS Stimulation in Mouse BMDMs. A) Heatmaps showing H3K27ac and P300 signal enrichment in LPS‐specific SEs from public datasets, used to identify active enhancer regions. B) Venn diagram illustrating the overlap between LPS‐activated enhancers at 6 h, LPS‐induced TAPEs at 1 h, and LPS‐activated SEs at 6 h. C) Functional enrichment analysis of Gene Ontology Biological Process (GOBP) terms for ABC‐ranked target genes associated with early (1 h) and late (6 h) LPS‐induced enhancers. D) Ranking plot of stitched SEs based on H3K27ac ChIP‐seq signal intensity. The TNF‐9 region (chr17_35209593‐35210594), highlighted in red, ranks at the ninth most prominent enhancer. E) Genome browser tracks showing the *Tnfα* SE located at chr17:35209593‐35210594 (TNF‐9), with elevated H3K27ac and P300 signals upon LPS treatment, indicating strong enhancer activation. F) Mean‐average (MA) plot of differential expression analysis of transcriptionally active putative enhancers (TAPEs) between UT and LPS‐stimulated conditions. G) RNA‐seq and CUT&RUN genome browser tracks showing increased eRNA transcription from the TNF‐9 region in BMDMs following 1 h of LPS treatment. H) Expression levels of TNF‐9 eRNA in BMDMs following LPS stimulation.

Macrophages are key mediators of chronic inflammation, and their dysregulation leads to persistent inflammatory responses, making them critical therapeutic targets. In this study, we aimed to identify eRNA‐producing enhancer regions with SE features in macrophages by leveraging publicly available epigenomic data. First, we defined LPS‐responsive enhancer elements using publicly available chromatin immunoprecipitation sequencing (ChIP‐seq) datasets for H3K4me1, H3K27ac, and p300 in mouse bone marrow‐derived macrophages (BMDMs) (**Figure** **1**A). To identify LPS‐specific enhancers, we selected p300 signal variability between control and LPS conditions, given its pronounced changes upon LPS stimulation (Figure S1A,B, Supporting Information). Differential p300 signal activity was predominantly upregulated following LPS treatment, consistent with prior findings^[^ ^29^ ^]^ (Figure S1C,D, Supporting Information). In total, we identified 2067 LPS‐specific enhancers, each characterized by a pre‐existing H3K4me1 mark before stimulation, indicating a primed state even in the absence of LPS. Upon LPS exposure, H3K27ac and p300 activity increased (Figure 1A).

Next, we refined these LPS‐responsive enhancers by assessing their capacity to produce eRNAs. Given that eRNA synthesis often precedes mRNA induction and chromatin remodeling events, including histone acetylation and p300 recruitment,^[^ ^30^ ^]^ we performed total RNA‐sequencing (RNA‐seq) on BMDMs stimulated with or without LPS for 1 h, an earlier time point than that used in public datasets. From these data, we identified 1058 transcriptionally active putative enhancers (TAPEs) exhibiting bi‐directional transcription from consensus enhancers.^[^ ^30^ ^]^ Notably, eRNA production across these TAPEs displayed a clear separation between control and LPS conditions, even at this early time point (Figure S1E,F, Supporting Information). To pinpoint key regulatory elements driving LPS‐induced gene expression, we intersected enhancers identified at 6 h post‐LPS stimulation with t1‐h TAPEs and the 6‐h SEs using the ROSE algorithm.^[^ ^9^, ^31^ ^]^ This integrated approach allowed us to identify enhancers that were activated early yet sustained high enrichment levels, highlighting their central role in the inflammatory response and revealing key regulators of transcriptional changes relevant to disease. Ultimately, we identified 62 candidate regulatory elements that met these criteria: enhancers that generate eRNAs and exhibit SE features in mouse macrophages, which were rapidly activated upon LPS treatment (Figure 1B and Figure S1G, Supporting Information). Functional analysis of these enhancers demonstrated a dynamic immune response to LPS. At 1 h, LPS activated specific pathways, including hypersensitivity and acute inflammatory responses, indicating an early, targeted immune activation. By 6 h, the immune response had expanded, showing significant enrichment in pathways related to immune cell recruitment and activation, indicative of a transition from early, targeted signaling to a more widespread inflammatory state (Figure 1C and Figure S1H,I, Supporting Information).

Among the LPS‐responsive, eRNA‐producing enhancers with SE features, the enhancer‐gene pair linking chr17:35209593‐35210594 and the *Tnfα* gene ranked ninth overall. Its association with lymphotoxin alpha (*Ltα*) ranked third based on activity‐by‐contact (ABC) scores (Table S1, Supporting Information). Notably, among all enhancers associated with *Tnfα*, chr17_35 209 593_35 210 594 showed the highest ABC score, highlighting its strong regulatory potential (Figure 1D,E). *Tnfα* expression is regulated by multiple enhancers, including HSS +3, HSS‐0.8, and HSS‐9, originally identified as a DNase I hypersensitivity site (DHS).^[^ ^32^ ^]^ By aligning enhancer coordinates, we found that the “chr17:35209593‐35210594; TNF‐9 region” corresponds to HSS‐9. RNA‐seq and CUT&RUN analyses revealed that this TNF‐9 region exhibited significantly increased eRNA production after 1 h of post‐LPS stimulation (Figure 1F), along with elevated H3K27ac signals (Figure 1G,H). Analysis of public cap analysis gene expression sequencing (CAGE‐seq) data confirmed that transcripts from this enhancer display the bidirectional transcription characteristic of eRNAs (Figure S2A, Supporting Information). Furthermore, eRNA expression was exclusively detected from the TNF‐9 region, with no signal observed at other known HSS sites (Figure S2B, Supporting Information). Based on its strong regulatory potential, highest ABC score for Tnfα, robust eRNA induction upon LPS stimulation, and enrichment of active chromatin marks (H3K27ac and p300), we selected the chr17:35209593–35210594 region (TNF‐9) as a representative example for further functional analysis.

### 2.2 Disruption of the TNF‐9 Enhancer Reveals eRNA‐Mediated Control of Tnfα in Mouse Macrophages

To investigate the function of the TNF‐9 region and its associated eRNA, we generated KO mice lacking the TNF‐9 enhancer (Figure S3A, Supporting Information). In these TNF‐9 KO mice, a 707 bp region (chr17:35212876–35213582, mm10 genome) located 9 kb upstream of the *Tnfα* transcription start site (TSS) was deleted (Figure S3B,C, Supporting Information). To determine whether deletion of the TNF‐9 region affects immune cell homeostasis or generation, we analyzed various immune cell populations under resting conditions. No major differences were observed in the cellular composition of TNF‐9 KO mice (Figure S3D–G, Supporting Information), suggesting that the TNF‐9 region is dispensable for the immune cell development and homeostasis.

**Figure 2:**
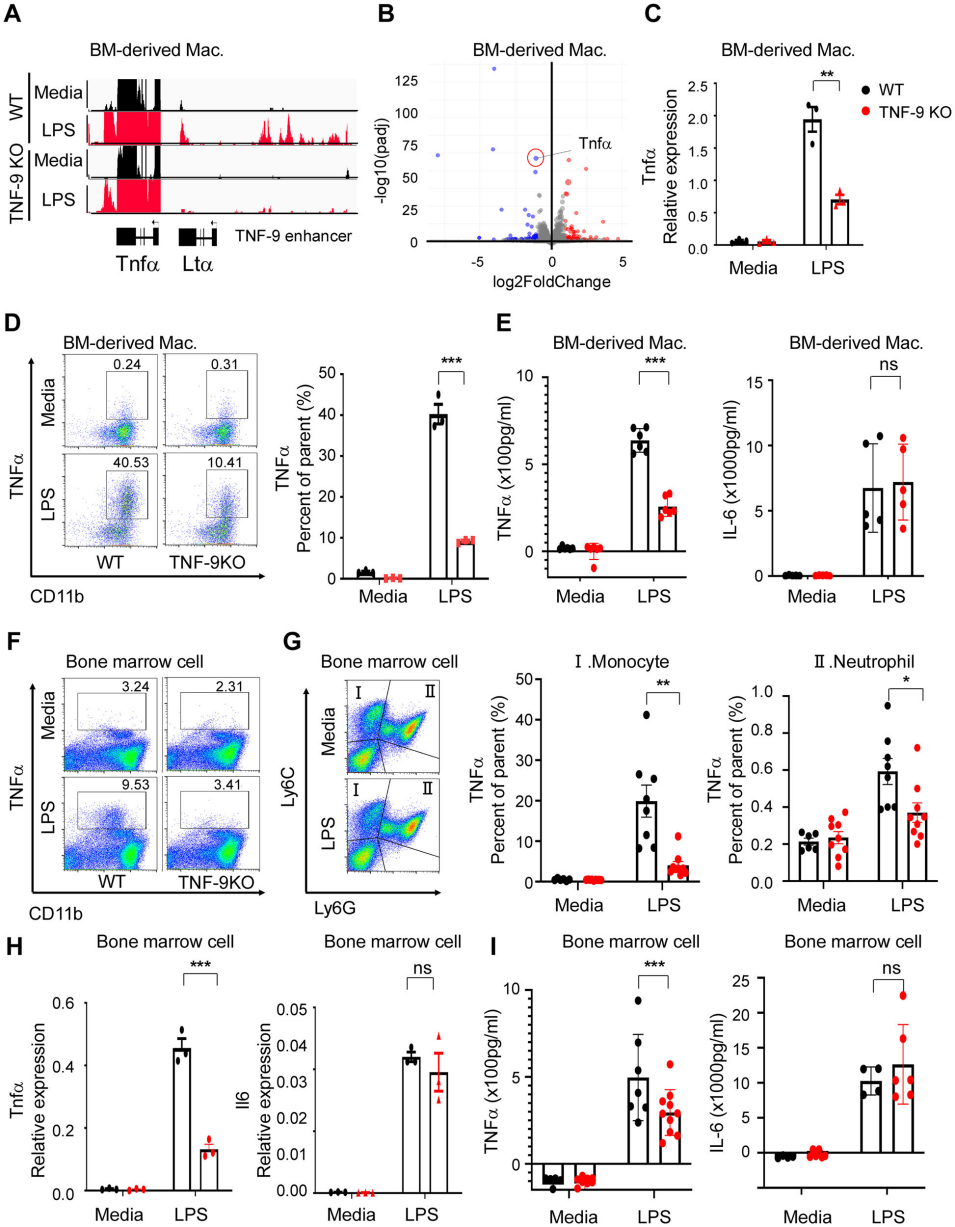
Deletion of TNF‐9 Impairs Tnfα Expression in Myeloid Cells. A) RNA‐seq peaks visualized using the Integrative Genome Viewer, highlighting the TNF‐9 region. B) Volcano plot comparing coding regions between LPS‐treated WT and TNF‐9 KO cells, highlighting DEGs. C) *Tnfα* expression changes after 1 h of LPS treatment in BMDMs. normalized to Gapdh. D) Representative flow cytometry plots showing a significant reduction in CD11b^+^ Tnfα^+^ cells in TNF‐9 KO mice compared to WT (left). Quantification of the percentage of CD11b^+^ Tnfα^+^ cells in BMDMs WT and TNF‐9 KO mice after LPS stimulation, shown as a bar graph (right). E) Tnfα and Il6 levels in culture supernatants from LPS‐stimulated BMDMs derived from WT and TNF‐9 KO mice. F) Percentage of CD11b^+^ Tnfα^+^ cells in LPS‐stimulated BM. Representative flow cytometry plots show a significant reduction in TNF‐9 KO mice compared to WT. G) Gating strategy used to identify specific populations, including Ly6C^+^ monocytes (Population I) and Ly6C^+^Ly6G^+^ neutrophils (Population II). The percentage of Tnfα^+^ cells was analyzed within each population and compared WT and TNF‐9 KO mice. H) *Tnfα* and *Il6* mRNA expression in BM from WT and TNF‐9 KO mice. I) Tnfα and Il6 levels in culture supernatants from LPS‐stimulated BM from WT and TNF‐9 KO mice. All data are presented as mean ± standard error of the mean (SEM). Statistical significance was determined using Student's *t*‐test (*p < 0.05, **p < 0.01, and ***p < 0.001).

Although TNFα is expressed in various immune cells, including T cells and macrophages,^[^ ^33^, ^34^, ^35^ ^]^ we focused on the TNF‐9 region in BMDMs. To examine the overall impact of the TNF‐9 region, we performed RNA‐seq on TNF‐9 KO and wild‐type (WT) BMDMs. Principal component analysis (PCA) revealed that PC1 primarily captured the transcriptomic differences between untreated (UT) and LPS‐stimulated cells, whereas PC2 distinguished WT from KO cells. Notably, LPS‐induced changes were more pronounced than those resulting from TNF‐9 KO (Figure S4A, Supporting Information). To investigate eRNA dynamics, we integrated a public assay for transposase‐accessible chromatin using sequencing (ATAC‐seq) data with our RNA‐seq analysis. In WT BMDMs stimulated with LPS for 6 h, a clear eRNA signal was detected at chr17:35209593‐35233637, indicating active transcription from this enhancer. In contrast, this TNF‐9 eRNA expression was undetectable in TNF‐9 KO BMDMs under the same conditions (Figure S4B, Supporting Information). LPS treatment significantly induced TNF‐9 eRNA expression in WT BMDMs, a response that was absent in TNF‐9 KO BMDMs (Figure S4C, Supporting Information). Consistently, genome browser views of our RNA‐seq data showed an upregulation of TNF‐9 eRNA expression in WT BMDMs following LPS treatment, which was abolished in TNF‐9 KO BMDMs (**Figure** **2**A).

Recent studies suggest that distal enhancers can regulate target genes over large genomic distances, even spanning different chromosomes.^[^ ^36^, ^37^ ^]^ To determine whether the TNF‐9 region influences genes beyond *Tnfα*, we conducted a detailed analysis of differentially expressed genes (DEGs). Although substantial differences were observed between UT and LPS‐treated conditions, the overall expression profiles of WT and TNF‐9 KO cells remained similar (Figure S4D, Supporting Information). However, within the set of LPS‐upregulated genes, *Tnfα* and a subset of other genes displayed differential expression between WT and KO cells (Figure 2B). Further filtering for coding genes confirmed that *Tnfα* expression was markedly reduced in TNF‐9 KO cells (Figure 2B,C). Consistent with the ABC score, *Ltα* expression was also diminished in TNF‐9 KO cells, whereas *Il6* and *Ltβ* remained unchanged (Figure S5A, Supporting Information). At the protein level, Tnfα expression was reduced in TNF‐9 KO cells, whereas Il6 levels remained unchanged (Figure 2D,E). Although the TNF‐9 region may regulate additional genes, our data suggest its effects are highly selective, primarily influencing *Tnfα* and a few immune‐related genes such as *Mid1* and *Ifi208* (Table S2, Supporting Information). Notably, most of these genes encode downstream signaling molecules of Tnfα, suggesting a potential autocrine mechanism involving Tnfα. Collectively, these findings highlight TNF‐9 as a crucial regulatory element for *Tnfα* expression, supported by the concomitant transcription of a distinct eRNA from this enhancer.

To further investigate the TNF‐9 enhancer function and its associated eRNA in myeloid cells, we harvested bone marrow from the femur and tibia of the WT and TNF‐9 KO mice, isolated bone marrow (BM) cells, and treated them with LPS. Deletion of TNF‐9 markedly reduced both TNF‐9 eRNA and *Tnfα* expression (Figure 2F). Because BM is heterogeneous, comprising hematopoietic progenitors, monocytes, and lymphocytes, we examined which cell types primarily produced Tnfα in response to LPS. Monocytes (Ly6C^+^Ly6G^−^) were identified as the primary Tnfα‐producing population, although neutrophils and other cell types contributed to some extent (Figure 2G and Figure S5B, Supporting Information). Consistent with this trend, Tnfα expression was significantly lower in TNF‐9 KO BM cells at both the mRNA (Figure 2H) and protein (Figure 2I) levels, whereas Il6 expression remained unchanged (Figure 2H,I). As previously observed, Ltα was also downregulated in TNF‐9 KO cells, while Ltβ levels remained unchanged (Figure S5C, Supporting Information). Overall, these results demonstrate that the TNF‐9 enhancer specifically regulates Tnfα and Ltα in response to LPS, underscoring its critical role in myeloid cell‐mediated inflammatory responses.

### 2.3 TNF‐9 Enhancer Deficiency Dampens Tnfα‐Driven Immune Activation in an LPS‐Induced Sepsis Model

Although anti‐TNF therapies are generally avoided in clinical sepsis due to risks of immunosuppression and secondary infections,^[^ ^38^ ^]^ our findings suggest that targeting specific regulatory elements like the TNF‐9 enhancer may provide a more precise means of attenuating early TNFα‐driven inflammation without broadly suppressing immune function. To further explore its systemic role in immune regulation, we utilized an LPS‐induced sepsis model. Following intravenous LPS injection, the percentages of Ly6C⁺Ly6G⁺ neutrophils in the blood of WT mice increased significantly (Figure S6A,B, Supporting Information). Additionally, Tnfα expression was markedly elevated in both Ly6C⁺ monocytes and Ly6C⁺Ly6G⁺ neutrophils, reflecting a robust response to LPS stimulation (Figure S6C, Supporting Information). In contrast, TNF‐9 KO mice exhibited significantly smaller increases in both Ly6C⁺ monocytes and Ly6C⁺Ly6G⁺ neutrophils, indicating impaired immune cell activation in the absence of the TNF‐9 enhancer (Figure S6D, Supporting Information). Consistent with these observations, serum Tnfα levels were significantly lower in TNF‐9 KO mice than that in WT mice, whereas Il6 levels remained unchanged (Figure S6E, Supporting Information). This selective reduction in Tnfα, but not Il6, indicates a specific regulatory role for the TNF‐9 enhancer in Tnfα expression and highlights its significance in immune cell activation during LPS‐induced sepsis. Collectively, these findings emphasize the broader physiological relevance of the TNF‐9 enhancer in modulating inflammatory responses under systemic inflammatory conditions.

### 2.4 Targeting the TNF‐9 Enhancer in RA and Psoriasis Demonstrates Broad Therapeutic Potential

**Figure 3:**
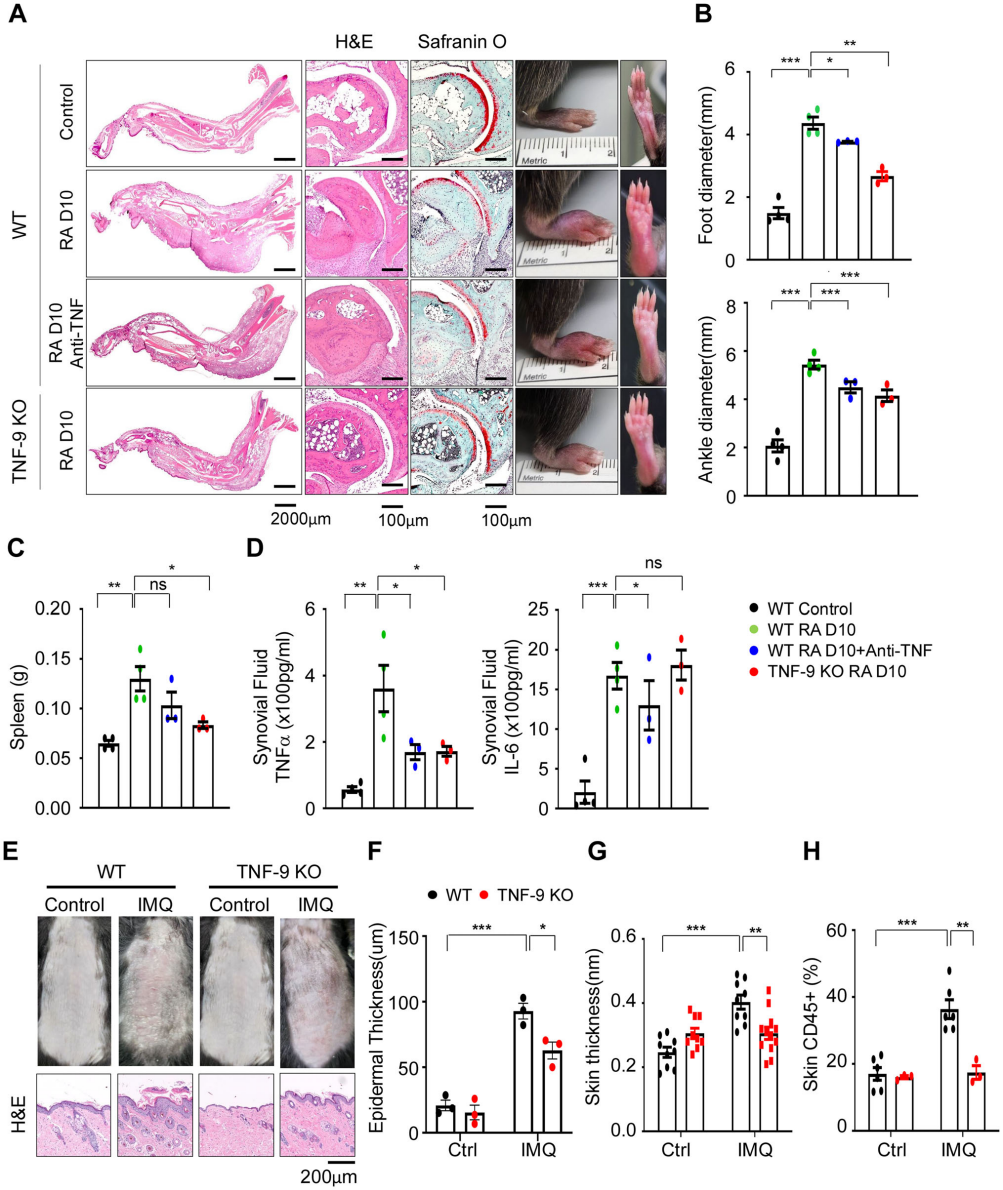
Deletion of the TNF‐9 Region Alleviates RA. A) Comparison of arthritis severity in AIA mouse models among WT, TNF‐9 KO, and WT mice treated with anti‐TNF. Representative hematoxylin and eosin staining and Safranin O staining of joint tissues in AIA mice at 10 day post‐induction. B) Measurements of foot diameter and ankle diameters in different mouse groups. C) Measurement of spleen weight in different mouse groups. D) Tnfα and Il6 levels in synovial fluid. E) Comparison of psoriasis symptoms between WT and TNF‐9 KO mice, assessed histologically. F,G) Measurement of epidermal and skin thickness. H) Flow cytometric analysis of CD45^+^ immune cell populations in the skin. Scale bars are indicated below each image. Data are presented as mean ± SEM. Statistical significance was determined using Student's *t*‐test (*p < 0.05, **p < 0.01, and ***p < 0.001).

We next sought to validate the role of the TNF‐9 enhancer in additional disease models mediated by Tnfα. Specifically, we employed two well‐established mouse models: the antigen‐induced arthritis (AIA) model for RA and the imiquimod (IMQ)‐induced psoriasis model, both of which are commonly treated with anti‐TNF therapies. In the AIA model, TNF‐9 KO mice exhibited significantly reduced joint swelling and erythema compared to WT mice (**Figure** **3**A). The decrease in clinical symptoms in TNF‐9 KO mice was comparable to the effects of anti‐TNF treatment^[^ ^39^ ^]^ (Figure 3A). Histological examination of decalcified joint tissue confirmed reduced inflammation in TNF‐9 KO mice (Figure 3A). Furthermore, measurements of ankle and foot diameters showed a pronounced reduction in swelling in TNF‐9 KO mice compared to that in WT mice (Figure 3B). Notably, TNF‐9 KO mice did not develop splenomegaly, an important indicator of systemic inflammation in the AIA model,^[^ ^40^ ^]^ whereas WT mice showed a significant increase in spleen weight 10 d post‐induction (Figure 3C). Synovial fluid analysis revealed a marked decrease in Tnfα levels in TNF‐9 KO mice, with Il6 levels remaining unchanged, consistent with previous observations (Figure 3D). Collectively, these findings demonstrate that TNF‐9 deletion mitigates RA severity by selectively modulating Tnfα expression, mirroring the therapeutic effects of anti‐TNF treatment.

In the IMQ‐induced psoriasis model^[^ ^41^, ^42^, ^43^ ^]^ (Figure S7A–C, Supporting Information), TNF‐9 KO mice exhibited markedly reduced psoriatic symptoms, including decreased skin thickness and inflammation compared to WT mice (Figure 3E,F). Histological analysis further revealed attenuated epidermal hyperplasia and reduced infiltration of CD45⁺ immune cells and neutrophils in the skin after 5 d of IMQ application (Figure 3G and Figure S7D, Supporting Information). Systemic analysis also showed reduced activation of neutrophils and monocytes in the blood of TNF‐9 KO mice, further supporting a diminished inflammatory response (Figure S7E, Supporting Information). TNF‐9 deletion significantly lowered Tnfα expression, improving RA and psoriasis symptoms. Therefore, TNF‐9 emerges as a promising therapeutic target for Tnfα‐mediated chronic inflammatory diseases across multiple organ systems.

### 2.5 ASO‐Mediated Knockdown of TNF‐9 eRNA Decreases Tnfα Depression in a Mouse Model of RA

**Figure 4:**
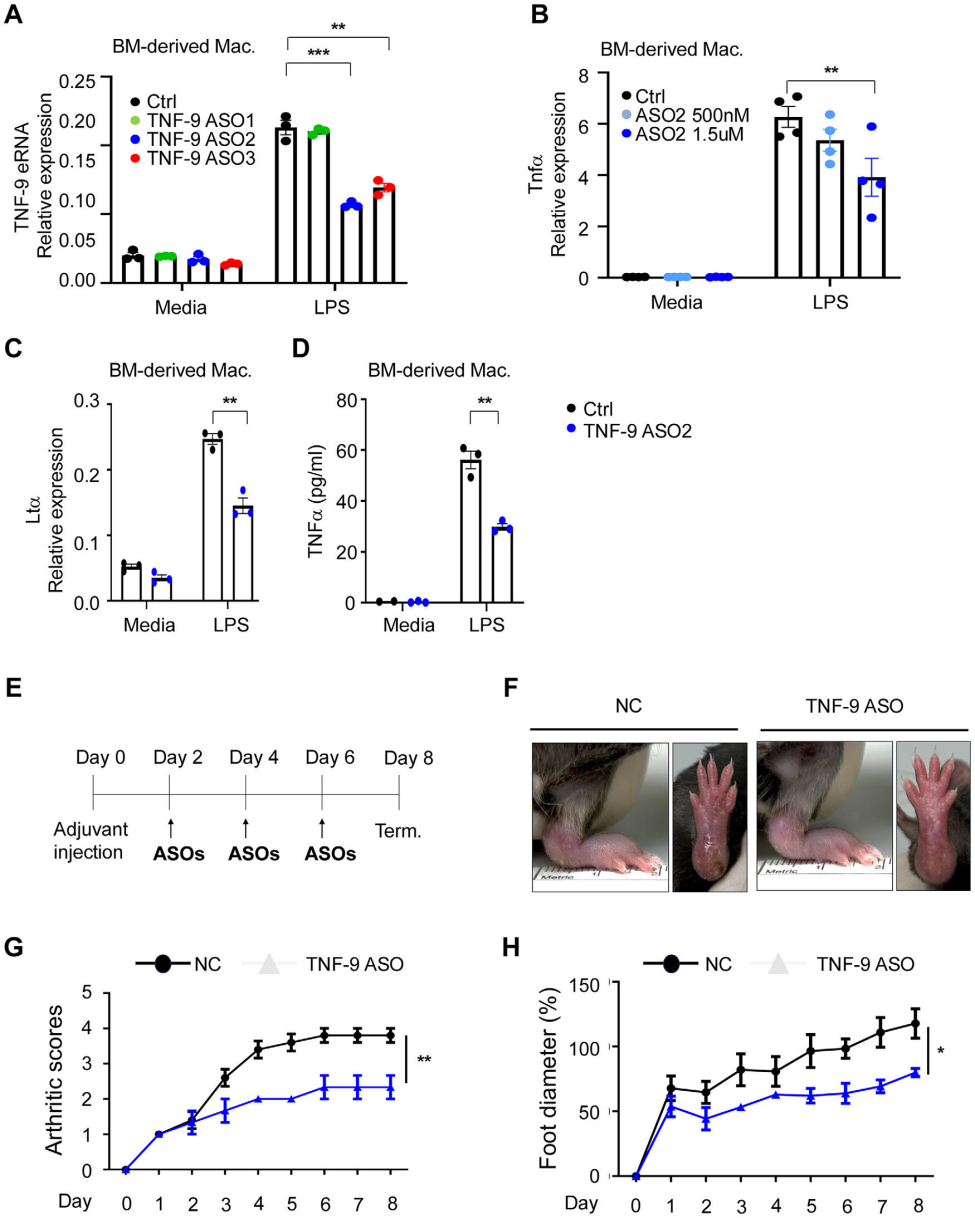
ASO‐Mediated Targeting of TNF‐9 eRNA Reduces *Tnfα* Expression. A) TNF‐9 eRNA expression following treatment with three different ASOs targeting TNF‐9 eRNA. B) Dose‐dependent effect of ASO2 concentration on Tnfα expression. C) Ltα expression in LPS‐treated BMDMs after ASO2 treatment. D) Tnfα levels in culture supernatants from LPS‐treated BMDMs after ASO2 treatment. E) Schematic of the AIA mouse model timeline and key experimental steps, including treatment administration, sample collection, and analysis points. F–H) Arthritic scores and foot diameter measurements in RA‐induced mice treated with ASO2. Data are presented as mean ± SEM. Statistical significance was determined using Student's *t*‐test (**p < 0.01 and ***p < 0.001).

Thus far, we have characterized the overall role of the TNF‐9 enhancer; however, we specifically aimed to modulate its eRNA function. To achieve this, we employed ASO treatment to knock down TNF‐9 eRNA in mouse BMDMs. Three different ASOs targeting TNF‐9 eRNA were designed and delivered via electroporation. TNF‐9 eRNA expression levels were significantly reduced after ASO treatment, with ASO2 exhibiting the highest knockdown efficiency (**Figure** **4**A). Consequently, ASO2 was chosen for further experiments. A dose‐dependent decrease in *Tnfα* expression was observed with increasing concentrations of ASO2 (Figure 4B). Since previous data confirmed that the TNF‐9 enhancer also regulates *Ltα*, we examined *Ltα* expression. Targeting TNF‐9 eRNA with ASO2 led to a significant reduction in *Ltα* mRNA (Figure 4C) and a corresponding decrease in Tnfα protein levels (Figure 4D). These findings demonstrate that TNF‐9 eRNA directly influences Tnfα and Ltα expression, underscoring its critical regulatory role in inflammatory signaling pathways.

To further validate the regulatory role of the TNF‐9 enhancer, we employed a CRISPR interference (CRISPRi) strategy using a dCas9‐KRAB system in mouse macrophage J774A.1 cells.^[^ ^44^ ^]^ Three guide RNAs (gRNAs) targeting the TNF‐9 locus were designed and transfected, followed by LPS stimulation. Among the tested gRNAs, gRNA2 showed the most pronounced suppressive effect. Repression of the TNF‐9 enhancer by gRNA2 led to a significant reduction in *Tnfα* gene expression and Tnfα protein levels, confirming the enhancer's functional involvement in LPS‐induced inflammatory signaling (Figure S8A,B, Supporting Information). These results are consistent with our ASO‐based findings and further support the role of TNF‐9 as a key regulatory element in Tnfα transcription.

We next evaluated the therapeutic potential of ASO‐mediated TNF‐9 eRNA knockdown in a mouse model of RA. ASO2 was administered intravenously following a defined dosing schedule (Figure 4E). Mice treated with ASO2 displayed significantly reduced joint swelling, as indicated by decreased foot and ankle diameters (Figure 4F–H). These data suggest that ASO‐based inhibition of TNF‐9 eRNA effectively reduces inflammation and alleviates disease symptoms. Collectively, our results establish ASO‐mediated TNF‐9 eRNA targeting as a promising strategy for regulating Tnfα expression and mitigating inflammatory disease pathology, including RA and psoriasis. This approach provides a targeted and potentially safer alternative to conventional anti‐TNF therapies.

### 2.6 Identification of DHS44500 as a Conserved Human TNFα Enhancer and Its Activation in RA and Psoriasis

**Figure 5:**
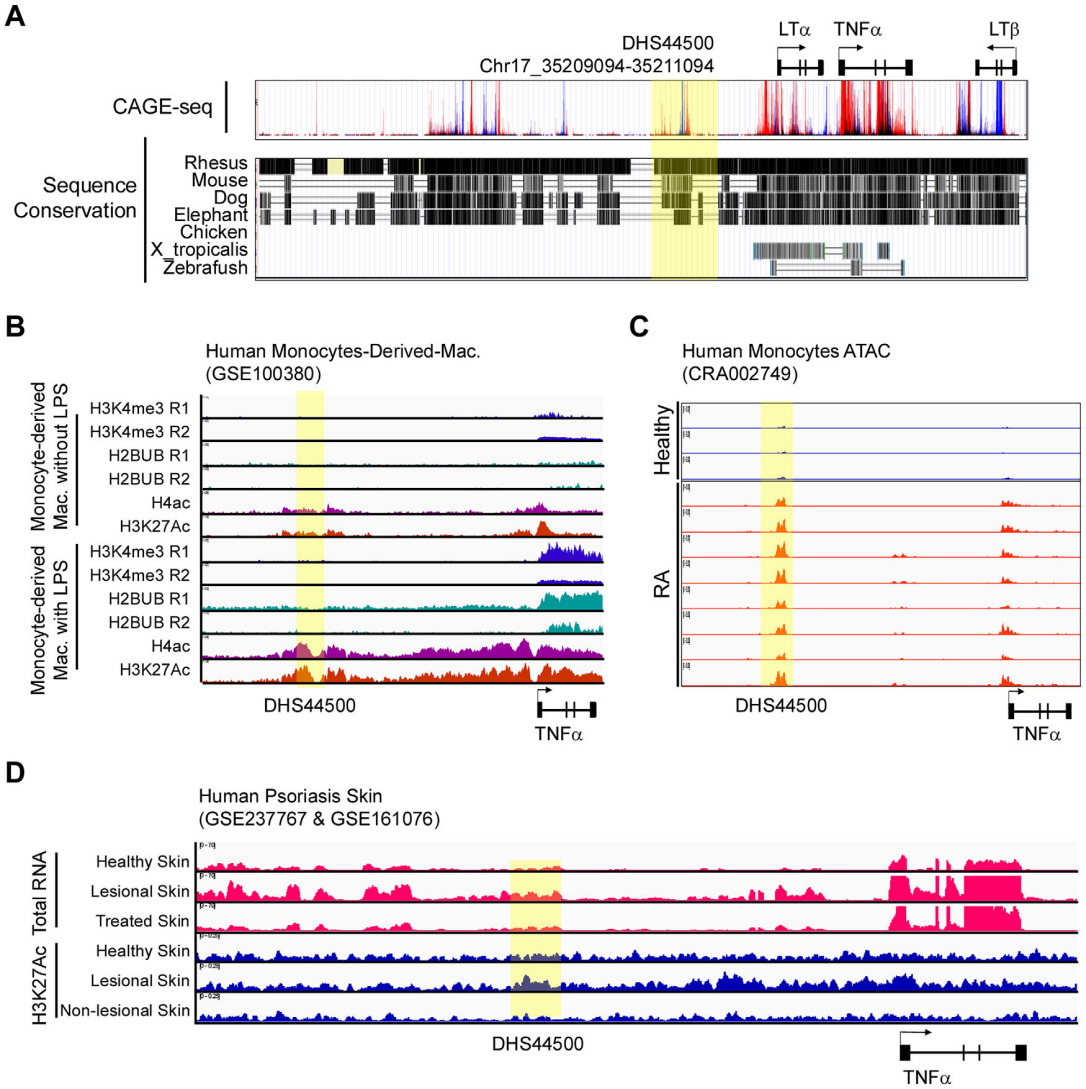
Comprehensive Analysis of the TNFα Enhancer Region, DHS44500, in Human Monocyte‐Derived Macrophages and Samples from Patients with RA. A) UCSC Genome Browser view showing CAGE‐seq data and Multiz Alignments of 100 Vertebrates, illustrating homology and sequence conservation. B) ChIP‐seq analysis of H3K4me3, H2Bub, H4ac, and H3K27ac in human monocyte‐derived macrophages before and after LPS treatment. C) ATAC‐seq data comparing chromatin accessibility at DHS44500 in monocytes from patients with RA and healthy controls. D) Total RNA‐seq and H3K27ac ChIP‐seq analysis of skin tissues from patients with psoriasis and healthy controls. The DHS44500 region is highlighted in a fluorescent yellow in all panels.

To extend our mouse‐based findings on the TNF‐9 enhancer to potential therapeutic applications in humans, we next sought to identify and characterize a homologous regulatory region in the human genome. Using the UCSC Genome Browser with Multiz Alignment of 100 Vertebrates and CAGE‐seq datasets, we identified DHS44500, a highly conserved human TNFα enhancer region analogous to the mouse TNF‐9. This element^[^ ^45^ ^]^ is conserved across multiple species, as shown by the Multiz alignments (**Figure** **5**A). The top panel of Figure 5A shows bidirectional transcription, a hallmark of eRNAs, with CAGE‐seq data revealing transcription peaks in both directions.

To investigate the role of DHS44500 in human immune responses, we examined its activity in LPS‐treated human monocyte‐derived macrophages (Figure 5B). Following LPS stimulation, DHS44500 displayed histone modifications characteristic of active enhancers, including increased H3K27ac and H4ac signals. To evaluate its relevance in disease, we analyzed public epigenomic datasets from patients with RA (CRA002749) and psoriasis (GSE237767 and GSE161076) (Figure 5C,D). In RA, chromatin accessibility at DHS44500 was elevated in monocyte‐derived macrophages from patients compared to healthy controls (Figure 5C). In psoriasis, although the data reflect whole tissue analyses, eRNA expression at DHS44500 was higher in lesional skin than in healthy skin and was reversed following treatment with calcipotriene and betamethasone dipropionate (Figure 5D). Similarly, H3K27ac signals were increased in lesional skin but resembled healthy controls in non‐lesional areas (Figure 5D). Collectively, these findings indicate that DHS44500 functions as an active enhancer not only in LPS‐induced in vitro conditions but also in human inflammatory diseases across multiple organs.

### 2.7 ASO‐Based Inhibition of DHS44500 eRNA Regulates TNFα Expression

**Figure 6:**
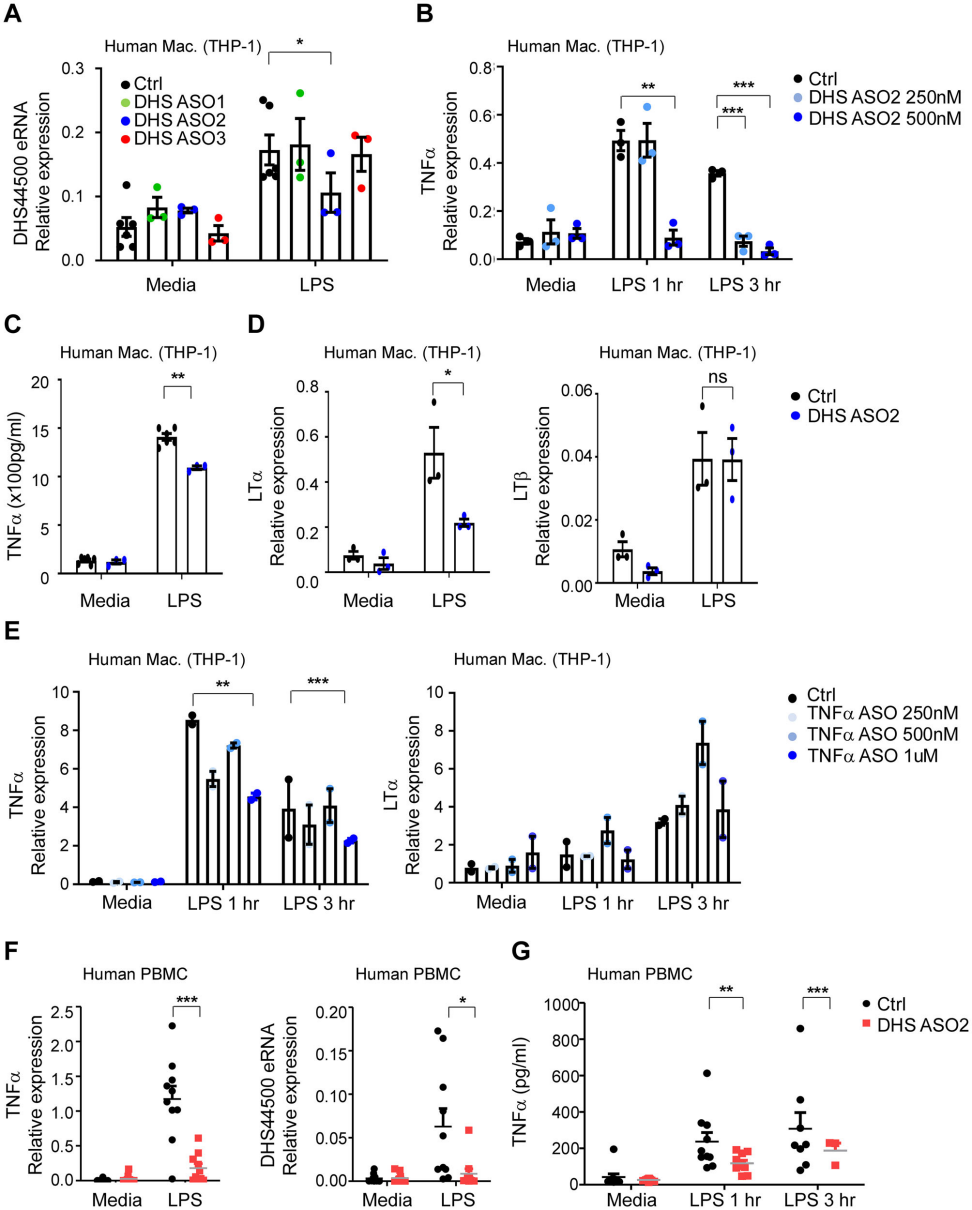
ASO Targeting of DHS44500 eRNA Modulates TNFα Expression in Human Cells. A) THP‐1 cells were electroporated with three candidate ASOs targeting DHS44500 eRNA, followed by LPS stimulation to induce cytokine expression. The expression level of DHS44500 eRNA was subsequently measured. B) Knockdown efficiency and dose‐dependent effects of ASO2 treatment were assessed by quantifying *TNFα* mRNA expression. C,D) Protein levels of TNFα (C) and LTα and LTβ (D) were evaluated following ASO2 treatment to determine its impact on cytokine expression. E) BMDMs were electroporated with ASOs targeting the TNFα coding region. After 24 h, the cells were stimulated with LPS for 1 and 3 h. The expression of TNFα and LTα was quantified. F) PBMCs were treated with ASO2 targeting DHS44500 eRNA to assess *TNFα* and DHS44500 expression. G) TNFα protein levels in PBMC culture supernatants after ASO2 treatment (n = 10). Data are presented as mean ± SEM. Statistical significance was determined using Student's *t*‐test (*p < 0.05, **p < 0.01, and ***p < 0.001).

While DHS44500 was identified as an enhancer activated under inflammatory conditions, including LPS stimulation, RA, and psoriasis, direct evidence linking it to human TNFα regulation was needed. To evaluate this connection between DHS44500 activation and TNFα upregulation, we designed an ASO targeting human DHS44500 eRNA. Three candidate ASOs were electroporated into human monocytic THP‐1 cells, and ASO2 was the most effective in reducing DHS44500 eRNA expression (**Figure** **6**A). Consistent with previous observations, ASO2 treatment led to a significant dose‐dependent decrease in TNFα expression at both mRNA and protein levels (Figure 6B,C). LTα expression was also reduced, whereas LTβ levels remained unchanged (Figure 6D).

To further validate the functional relevance of DHS44500, a CRISPRi approach was employed using a dCas9‐KRAB system in human monocytic U937 cells. Three gRNAs targeting the DHS44500 locus were designed and transfected, followed by LPS stimulation. Among the candidates, gRNA1 exhibited the most substantial suppressive effect, resulting in a significant reduction of TNFα mRNA and protein levels (Figure S8C,D, Supporting Information). These results independently confirm the regulatory role of DHS44500 in inflammatory gene expression and are consistent with our ASO‐based findings, underscoring the enhancer's contribution to TNFα transcriptional control.

To validate the advantage of targeting the enhancer instead of the TNFα coding sequence, we performed ASO‐mediated knockdown of *TNFα* mRNA. While this approach effectively lowered *TNFα* expression (Figure 6E), it had no impact on LTα expression or DHS44500 eRNA levels (Figure 6E and Figure S9A, Supporting Information). Given that enhancers can regulate multiple genes, as shown by our ABC score analysis, we targeted the DHS44500 enhancer rather than the TNFα coding region, as it allows modulation of functionally related cytokines such as LTα, which plays a critical role in specific disease contexts. Collectively, these findings underscore the potential of eRNA‐targeted ASO therapy as a novel strategy for fine‐tuning cytokine expression networks, offering broader therapeutic effects beyond individual cytokines.

Since TNF‐9 eRNA targeting effectively modulated TNFα expression and alleviated RA symptoms in mice, we next investigated whether a similar approach could be applied to human cells by targeting DHS44500 eRNA. To this end, peripheral blood mononuclear cells (PBMCs) were isolated from the blood of patients with RA and stimulated to induce an immune response. This led to a substantial increase in *TNFα* expression along with an upregulation of DHS44500 eRNA (Figure 6F and Figure S9B, Supporting Information). Treatment with an ASO designed to target DHS44500 eRNA resulted in a significant decrease in the expression of *TNFα*, *LTα*, and DHS44500 eRNA (Figure 6F and Figure S9B, Supporting Information), with a corresponding decrease in TNFα protein levels (Figure 6G). These findings demonstrate that DHS44500 and its associated eRNA play a pivotal role in regulating TNFα expression in human PBMCs, mirroring the effects observed in mice.

### 2.8 Identification of LPS‐Responsive eRNA‐Producing SEs in Human Monocytes and Macrophages as Potential Therapeutic Targets

We identified and validated the TNF‐9 enhancer in mice and its human homolog, DHS44500, as a regulator of TNFα with potential for ASO‐based therapeutic targeting. Building on these findings, we applied the same analytical strategy used in our mouse studies to publicly available transcriptomic and epigenomic datasets, aiming to define eRNA‐producing enhancers with SE features in human monocytes and macrophages. Our goal was to identify a comprehensive list of potential ASO targets that may offer novel therapeutic options for chronic inflammatory conditions.

**Figure 7:**
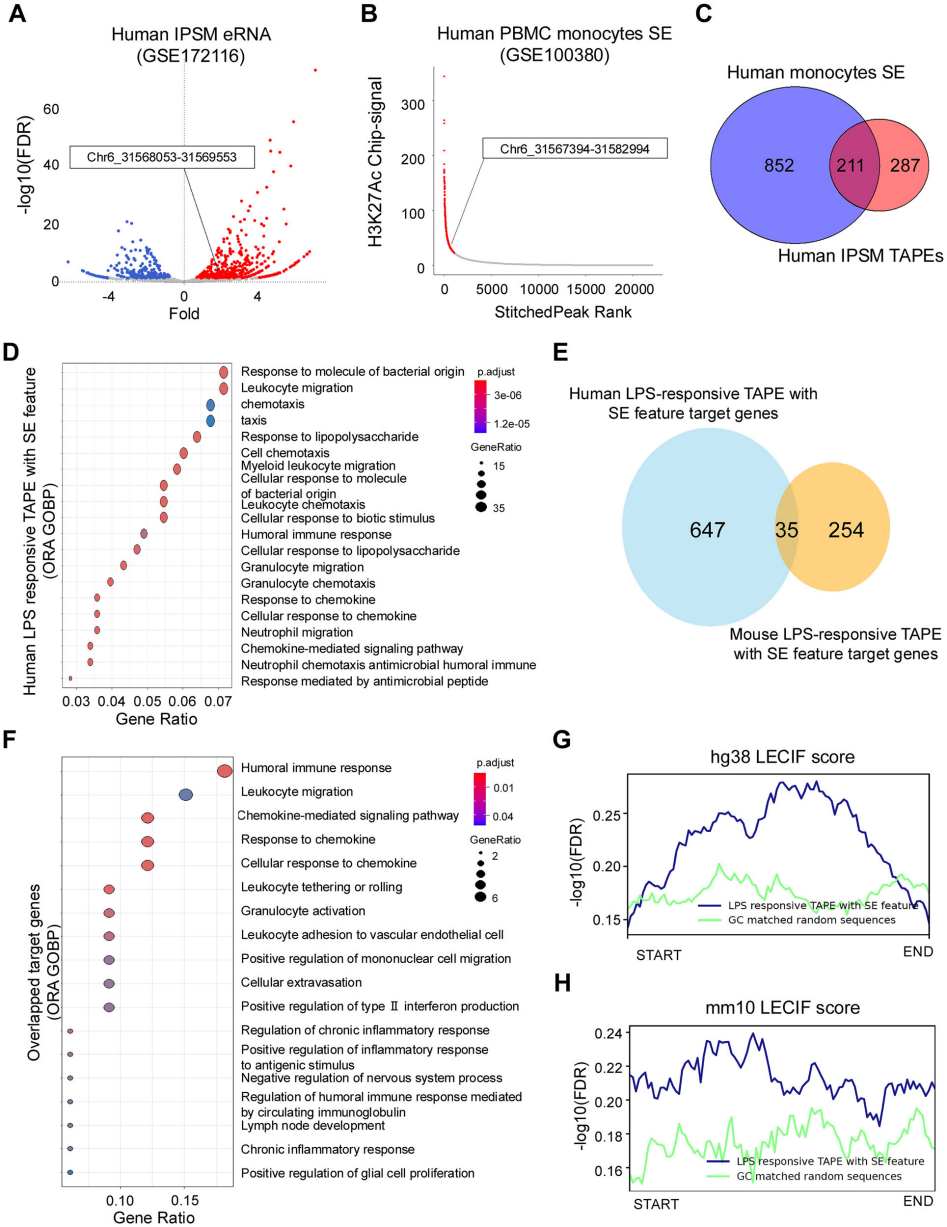
Evolutionarily Conserved SE‐Associated eRNAs Mediate LPS‐Induced Transcriptional Responses in Human and Mouse Monocytes. A) Volcano plot of differentially expressed eRNAs from TAPE analysis of human iPSM RNA‐seq data, comparing LPS‐stimulated and unstimulated conditions. The DHS44500 region (chr6:31568053‐31569553) is highlighted. B) Ranking plot of stitched SEs based on H3K27ac ChIP‐seq signal intensity in human PBMC‐derived monocytes. The DHS44500 region is highlighted. C) Venn diagram showing the overlap between human monocyte‐derived SEs and human iPSM‐derived TAPEs. D) Functional enrichment analysis of GOBP terms for ABC‐ranked target genes associated with human LPS‐responsive eRNA‐producing enhancer with SE features. E) Venn diagram illustrating the overlap of target genes between human and mouse LPS‐responsive TAPEs with SE features. F) Pathway enrichment analysis of GOBP terms for shared target genes of human and mouse LPS‐responsive TAPEs with SE features. G–H) Comparative analysis of LECIF scores for LPS‐responsive TAPEs with SE features versus GC‐matched random sequences in the hg38 (G) and mm10 (H) genomes.

Using public RNA‐seq data from LPS‐treated induced pluripotent stem cell‐derived macrophages (iPSMs; GSE172116) and ATAC‐seq data from LPS‐treated human monocytes (GSE100380), we identified 498 LPS‐responsive TAPEs (**Figure** **7**A). Next, by analyzing H3K27ac ChIP‐seq data from LPS‐treated monocytes (GSE100380), we identified 366 SE regions (Figure 7B). Notably, DHS44500 was present in both datasets (Figure 7A,B). Intersecting these datasets revealed 211 eRNA‐producing enhancers with SE features (Figure 7C). Target gene predictions using the ABC model showed enrichment for immune‐related functions, reflecting diverse immunological processes (Figure 7D). Among these, pathways involved in early immune responses, such as chemotaxis, myeloid leukocyte migration, and response to chemokines, were prominently represented. We compiled a list of genes potentially linked to these eRNA‐producing enhancers with SE features (Table S3, Supporting Information), suggesting that further investigation of these eRNAs could provide additional regulatory insights and potential therapeutic targets for chronic inflammatory diseases.

To assess the conservation of eRNA‐producing enhancers with SE features between humans and mice, we compared their target genes across both genomes. We identified 35 overlapping LPS‐responsive target genes (Figure 7E), predominantly involved in chemokine signaling pathways and leukocyte migration (Figure 7F). Additionally, we used the Learning Evidence of Conservation from Integrated Functional (LECIF) genome annotation scores^[^ ^46^ ^]^ to quantify human–mouse sequence conservation. The LPS‐responsive eRNA‐producing enhancers with SE features consistently showed higher LECIF scores than GC‐matched random sequences (Figure 7G,H). These findings indicate that LPS‐responsive enhancers producing eRNAs and exhibiting SE characteristics are conserved between humans and mice at both sequence and functional levels. This conservation underscores their potential as therapeutic targets and supports the translational relevance of our preclinical findings to human biology.

## 3 Discussion

In this study, we investigated the regulatory role of eRNAs associated with SEs in controlling TNFα expression. Our findings demonstrate that targeting the eRNA transcribed from the TNF‐9 region in mice, as well as its homologous DHS44500 region in humans, effectively reduces TNFα levels.

TNFα is a critical pro‐inflammatory cytokine that plays a pivotal role in various immune responses, including the development of psoriasis, autoimmune diseases, and chronic inflammation.^[^ ^47^ ^]^ While anti‐TNF therapies have demonstrated efficacy in various chronic inflammatory diseases,^[^ ^48^ ^]^ they face significant limitations, including high costs, prolonged development timelines, and incomplete patient responsiveness.^[^ ^49^, ^50^ ^]^ Additional concerns include the risk of latent infections such as tuberculosis and the need to co‐administer disease‐modifying antirheumatic drugs to enhance efficacy.^[^ ^51^ ^]^ Moreover, anti‐TNF biologics induce broad immunosuppression, potentially increasing infection risks and compromising baseline immune function. To address these challenges, we explored ASOs as a therapeutic strategy for regulating TNFα. ASOs offer several advantages over biologics, including faster development timelines and patient‐specific customization.^[^ ^52^ ^]^ By directly binding to RNA, ASOs provide precise control over TNFα expression without interfering with upstream or downstream immune pathways. In contrast to anti‐TNF antibodies, which either bind soluble TNFα or block TNF receptors, our TNF‐9/DHS44500‐targeting ASOs selectively suppress TNFα transcription only when their expression surpasses homeostatic levels, thereby minimizing the risk of excessive immunosuppression. This targeted mechanism offers a safer and more refined alternative to conventional anti‐TNF therapies.

Despite the therapeutic promise of enhancer‐targeted ASO strategies, several limitations of our study should be acknowledged. First, although ASOs were designed with high sequence specificity, the possibility of off‐target effects cannot be entirely excluded and warrants further investigation through transcriptome‐wide analyses. In addition, while our results demonstrate consistent effects across RA, psoriasis, and sepsis models, additional in vivo validation in other disease settings and with varied dosing regimens will be essential to assess the long‐term safety, tissue specificity, and translational applicability of this approach. Addressing these limitations will be critical for advancing eRNA‐directed therapeutics toward clinical development.

Our results suggest that TNF‐9 eRNA targeted therapy may also regulate LTα expression, another cytokine implicated in various inflammatory processes, including ocular inflammation.^[^ ^53^, ^54^, ^55^ ^]^ Expanding eRNA‐targeting strategy to include LTα could further broaden their therapeutic applicability to chronic inflammatory diseases. By simultaneously modulating multiple pro‐inflammatory mediators, eRNA‐targeted therapies hold promise for achieving more comprehensive disease control.

Our transcriptomic analyses were conducted at an early time point (1 h post‐LPS stimulation) to capture primary enhancer activity and minimize confounding secondary effects. This decision was guided by prior studies showing that eRNAs represent some of the earliest transcriptional events following immune stimulation, often preceding mRNA accumulation of their associated genes.^[^ ^56^ ^]^ By selecting this early window, we aimed to dissect primary eRNA‐driven regulatory programs before the onset of widespread feedback signaling, chromatin remodeling, or secondary transcriptional waves.

**Figure 8:**
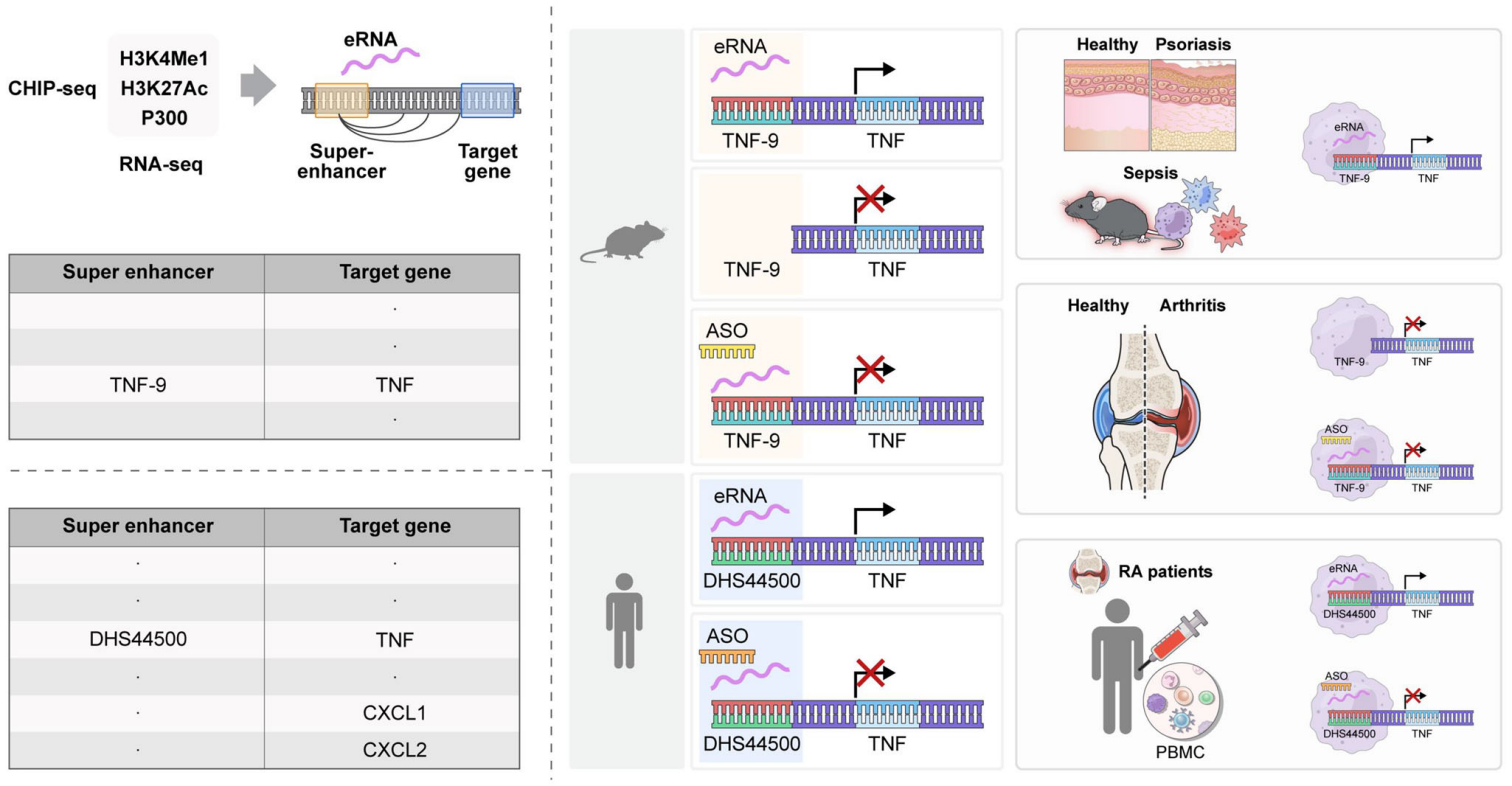
Graphical abstract of the super‐enhancer eRNA‐targeting therapeutic strategy for inflammatory diseases. Illustration of the regulatory role of SE‐associated eRNAs in controlling TNFα expression in murine and human immune cells. ChIP‐seq and RNA‐seq analyses identify TNF‐9 (mouse) and DHS44500 (human) as key SE that drive TNFα transcription. ASO therapy effectively suppresses TNFα expression by targeting these eRNAs and disrupting enhancer‐promoter interactions. Dysregulation of these eRNAs is implicated in inflammatory diseases, including psoriasis, sepsis, and RA. In RA patients, PBMCs stimulated with LPS show elevated expression of DHS44500 eRNA and TNFα, and ASO‐mediated knockdown of DHS44500 eRNA reduces TNFα levels. These findings establish eRNA‐targeted interventions as a promising therapeutic approach for chronic inflammatory diseases.

In particular, TNF‐9 KO mice exhibited improved clinical outcomes in RA and psoriasis models, two TNFα‐driven chronic inflammatory diseases, manifesting as reduced joint swelling, diminished psoriatic lesions, and lower overall immune cell activation. These results underscore the critical role of TNF‐9 in orchestrating TNFα‐driven inflammation across multiple tissues. A graphical summary of our findings is provided in **Figure** **8**, illustrating the role of eRNA‐producing SE in TNFα regulation and their therapeutic potential. Additionally, TNF‐9 KO mice displayed reduced Tnfα production and attenuated immune cell activation in various mouse model, reinforcing the broader therapeutic potential of eRNA‐targeted strategies.

The translational relevance of our findings was confirmed through experiments using PBMCs from patients with RA. Upon immune stimulation, these cells exhibited elevated TNFα levels alongside increased expression of DHS44500 eRNA, paralleling our findings in the mouse model. ASO‐mediated knockdown of DHS44500 eRNA significantly reduced TNFα levels in human cells, underscoring the functional conservation of TNF‐9 in mice and DHS44500 in humans. Notably, LECIF score analysis revealed that eRNA‐producing enhancers with SE features exhibit higher sequence conservation than random genomic regions, further supporting their translational potential. Moreover, the therapeutic efficacy of this approach in a psoriasis model suggests that eRNA‐targeting ASOs could benefit a broad spectrum of TNF‐driven pathologies.

Beyond TNF‐9, we identified additional eRNA‐producing SEs that may regulate key inflammatory genes (Table S3, Supporting Information). For example, KYNU and CCL2, involved in macrophage polarization and synovial inflammation, respectively, are associated with multiple eRNAs.^[^ ^57^, ^58^, ^59^, ^60^ ^]^ Further research into these enhancers could deepen our understanding of how eRNAs orchestrate complex inflammatory networks and lead to more effective therapeutic approaches. Such investigations would benefit from functional screening approaches targeting multiple enhancers, which could potentially reveal whether combinatorial eRNA modulation offers enhanced therapeutic efficacy while maintaining target specificity.

While our findings establish a clear link between eRNA‐producing SEs and TNFα regulation, several key research directions warrant further exploration. First, optimizing ASO delivery and stability could significantly enhance therapeutic efficacy. For instance, advanced nanoparticle carriers or cell‐specific targeting ligands may help minimize off‐target effects and improve tissue specificity. Second, investigating the synergistic potential between eRNA‐targeted therapies and existing biologics may uncover combinatorial strategies that achieve more robust and sustained disease control. Third, detailed mechanistic studies of additional eRNA‐producing enhancers could clarify how multiple eRNAs converge on shared inflammatory pathways. This knowledge may ultimately guide the development of multi‐eRNA targeting regimens that modulate complex cytokine networks. Finally, future research should evaluate the long‐term safety and immunogenicity of repeated ASO administration, particularly in chronic disease settings. Addressing these challenges will be crucial for translating eRNA‐targeted approaches from preclinical models to clinical applications, ultimately maximizing their potential as precision therapies for chronic inflammatory diseases.

In conclusion, our study highlights the pivotal role of SE‐derived eRNAs in regulating TNFα and establishes these molecules as promising therapeutic targets for chronic inflammatory diseases, including RA, psoriasis, and sepsis. While further research is required to refine ASO design, optimize delivery methods, and explore synergies with existing therapies, our findings provide the first demonstration that eRNA‐targeted strategies can be effectively harnessed to treat RA and psoriasis. This represents a major step toward developing safer and more precise immunomodulatory therapies. By leveraging the specificity of ASOs, we can potentially limit adverse effects while preserving essential immune functions. Taken together, our results position eRNA‐producing enhancers with SE features as valuable candidates for translational research, paving the way for innovative drug development and advancing precision medicine in the management of chronic inflammatory conditions.

## 4 Experimental Section

### Mouse Strains

All animals used in this study were housed in specific pathogen‐free conditions with ad libitum access to food and water at the Animal Resource Center of Yonsei University (Seoul, Korea). The study was conducted under Institutional Animal Care and Use Committee protocol (no. 2019‐0163) approved by Yonsei University College of Medicine. Mice were maintained under a 12‐h light/12‐h dark cycle with humidity levels between 40 and 60%, temperature maintained at 22± 2 °C. Only male mice aged 7–10 weeks old were used in this study. C57BL/6 mice were purchased from Orient Bio (Seoul, Korea). TNF‐9 KO mice were maintained as homozygous (KO/KO) breeding pairs to ensure that all offspring carried the homozygous deletion. Genotyping was performed on tail tips using a standard PCR‐based protocol to confirm the deletion. Pups were weaned at 21 d of age and separated by sex.

### BM Isolation and BMDM Culture

BMs were extracted from the femur and tibia of mice. To generate BMDMs, BM cells were cultured in Dulbecco's Modified Eagle's Medium (HyClone, SH30243.01) supplemented with 10% fetal bovine serum (FBS; Gibco, 10 099 141) and 20% L929‐conditioned media. After 6 d in culture, cells were seeded and treated under the indicated conditions.

### LPS‐Induced Sepsis, AIA, and IMQ‐Induced Psoriasis Mouse Models

To induce sepsis, mice were injected intravenously with 100 µg of LPS (Sigma‐Aldrich, L4130) dissolved in 100 µL of phosphate‐buffered saline (PBS; HyClone, SH30028.FS). Blood samples were collected 3 h post‐injection for subsequent analysis. To induce arthritis, 8‐week‐old mice received an injection of 100 µL of complete Freund's adjuvant (Sigma‐Aldrich, 7009) into the right hind paw. Disease progression was monitored daily by measuring ankle and paw thickness using digital calipers to quantify swelling and inflammation. On day 8 post‐injection, mice were euthanized via CO_2_ inhalation, followed by cervical dislocation. Blood samples were collected, and tissues, including affected joints, were harvested for histological and molecular analyses. To induce psoriasis, the dorsal skin of 8‐week‐old mice was shaved 48–72 h before the start of the experiment. IMQ cream (5%; Aldara) was applied daily to the shaved area to induce psoriasis‐like skin inflammation. On day 5 of the IMQ application, mice were euthanized using CO_2_ inhalation, followed by cervical dislocation. Full‐thickness dorsal skin samples and blood were collected for downstream analyses.

### Histopathological Analysis

For histological evaluation of joint tissues, the right hind limb was collected from each mouse and fixed in 10% neutral‐buffered formalin for 48 h. The samples were then decalcified in 10% EDTA (pH 7.4) for 2 weeks, with regular solution changes. Decalcified tissues were processed, embedded in paraffin, and sectioned at 5 µm thickness using a microtome. Sections were stained with hematoxylin and eosin (H&E) following standard protocols to evaluate synovial hyperplasia, inflammatory cell infiltration, and bone erosion. To assess cartilage integrity, sections were further stained with Safranin O. After dewaxing and rehydration, slides were stained with Weigert's hematoxylin, Fast Green FCF, and Safranin O to visualize proteoglycan distribution within the cartilage. Skin tissue samples were fixed in 10% neutral‐buffered formalin for 48 h, embedded in paraffin, and sectioned. Sections were stained with H&E to evaluate epidermal thickening, immune cell infiltration, and psoriasis‐like morphological changes. All stained slides were scanned using the Axioscan7 digital slide scanner (Carl Zeiss, Germany) for high‐resolution imaging.

### Bulk RNA‐Seq Library Preparation and Pre‐Processing

RNA‐seq was performed using paired‐end sequencing on the Illumina HiSeq 3000 platform. Libraries were prepared using the TruSeq Stranded Total RNA Library Prep Globin Kit, following the manufacturer's instructions. Sequencing yielded paired‐end reads with a read length of 101 bp. Prepared bulk RNA‐seq library quality was checked using FastQC (v0.11.9). Reads were aligned to the GRCm38 reference genome using HISAT2 (v2.1.0).^[^ ^61^ ^]^ The resulting SAM files were converted to BAM files and indexed using SAMtools (v1.17).^[^ ^62^ ^]^ A count matrix was constructed using featureCounts (v2.0.0).^[^ ^63^ ^]^ BigWig tracks for individual libraries were generated using bamCoverage (v3.5.2). DEGs were identified using DESeq2 (v1.42.0) after filtering out genes with low read counts. Pairwise comparisons between conditions were performed, and DEGs were selected based on the following criteria: |log_2_FoldChange(FC)| > 1 and adjusted *p* < 0.05. Heatmaps were generated using the identified DEGs, with hierarchical clustering performed to organize expression profiles. To determine whether eRNAs were differentially expressed under LPS stimulation, BAM files from RNA‐seq analysis were overlapped with TAPEs to assess coverage at enhancer regions. Differential expression between conditions was determined using the criteria: |log_2_FC| > 2 and adjusted *p* < 0.1. Visualization of differential expression was performed using MA plots.

### CUT&RUN Library Preparation and Pre‐Processing

We performed CUT&RUN on BMDMs using the CUTANA ChIC/CUT&RUN Kit (EpiCypher, 14–1048) and the CUTANA CUT&RUN Library Prep Kit (EpiCypher, 14–1001), following the manufacturer's instructions. ≈1 × 10^5^ cells were prepared and immobilized on Concanavalin A‐coated magnetic beads. Cells were incubated with H3K4me1 (EpiCypher, 13–0057) and H3K27ac (EpiCypher, 13–0045) antibodies, followed by treatment with pAG‐MNase for targeted chromatin cleavage at 37 °C. The cleaved chromatin fragments were purified, and ≈5 ng of DNA was used for library preparation. End repair, A‐tailing, adapter ligation, and uracil excision were performed according to the CUTANA CUT&RUN Library Prep Kit protocol, followed by dual‐indexed PCR amplification using i5 and i7 primers. The libraries were purified and size‐selected using AMPure XP beads to remove contaminants and enrich for fragments of the desired size. Sequencing was performed on an Illumina NovaSeq 6000 platform using paired‐end (2 × 150 bp) reads, ensuring high coverage for downstream CUT&RUN analysis. The prepared CUT&RUN libraries were pre‐processed with the nf‐core CUT&RUN pipeline (v3.1),^[^ ^64^ ^]^ executed via Nextflow (v23.04.5) to ensure reproducible and standardized data analysis. The GRCm38 mouse genome assembly from GENCODE^[^ ^65^ ^]^ was used as the reference, along with its corresponding annotation file (GENCODE VM23) and primary assembly sequence. Raw sequencing reads were trimmed, quality controlled, and aligned to the reference genome using Bowtie2 (v2.4.4).^[^ ^66^ ^]^ To remove potential artifacts, regions overlapping a pre‐defined blacklist (mm10‐blacklist.v2) were excluded.^[^ ^67^ ^]^ Peaks were identified using MACS2 (v2.2.7.1) with a genome size of 1.87 × 10⁹ bp. Normalization of coverage was performed using counts per million (CPM) with a bin size of 1. Quality control and summary metrics were assessed using FastQC (v0.11.9) and deepTools (v3.5.1).^[^ ^68^ ^]^ The final processed data, including normalized coverage tracks, peak calls, and quality control metrics, were used for downstream analysis.

### Publicly Available Mouse and Human Dataset Acquisition and Pre‐Processing

Publicly available datasets were utilized for comprehensive epigenomic and transcriptomic analyses across mouse and human models of macrophage activation and inflammatory disease. For mouse BMDMs, ChIP‐seq datasets for H3K4me1, H3K27ac, and P300, as well as bulk RNA‐seq datasets before and after LPS stimulation, were obtained from GSE163293. In addition, ATAC‐seq datasets of mouse monocyte‐derived macrophages stimulated with LPS were retrieved from GSE222804 to investigate chromatin accessibility changes upon activation.

For human macrophage studies, bulk ATAC‐seq and ChIP‐seq datasets for H3K4me3, H2Bub, H4ac, and H3K27ac from monocyte‐derived macrophages before and after LPS stimulation were accessed from GSE100380. RNA‐seq datasets for iPSMs before and after LPS stimulation were retrieved from GSE172116. To examine disease‐specific chromatin states, ATAC‐seq datasets from blood monocytes of patients with RA and healthy controls were obtained from CRA002749, while RNA‐seq and H3K27ac ChIP‐seq datasets from psoriasis skin samples were sourced from GSE237767 and GSE161076, respectively.

The publicly available mouse and human ChIP‐seq datasets were processed using the nf‐core/chipseq pipeline (v2.0.0)^[^ ^64^ ^]^ executed via Nextflow (v23.04.3). The GRCm38 mouse genome assembly and GRCh38 human genome assembly from GENCODE^[^ ^65^ ^]^ were used as references. Raw sequencing reads were trimmed using Trim Galore (v0.6.7) with cutadapt (v3.4), followed by quality control with FastQC (v0.11.9). Trimmed reads were aligned to the reference genome using Bowtie2 (v2.4.4). Alignment statistics and filtering were performed using SAMtools (v1.15.1). For peak calling, MACS2 (v2.2.7.1) was used. In the case of the GSE100380 dataset for H3K27ac ChIP‐seq, owing to the lack of replicates and input (IgG) controls, peak files were directly downloaded from the GEO dataset for downstream analysis, with conversion from hg19 to hg38 using UCSC Genome Browser's LiftOver tool.^[^ ^69^ ^]^ BigWig tracks were generated using bamCoverage (v3.5.2) for individual libraries. The publicly available mouse and human RNA‐seq datasets were pre‐processed as described in the “Bulk RNA‐seq library preparation and pre‐processing” section, using the appropriate reference genome (mouse: GRCm38; human: GRCh38). The publicly available mouse and human ATAC‐seq datasets were processed with the PEPATAC pipeline (v0.11.3).^[^ ^70^, ^71^ ^]^ Briefly, raw sequencing reads were trimmed with Trim Galore (v0.6.7) and quality‐checked using FastQC (v0.11.9). Reads were aligned to the reference genome with Bowtie2 (v2.4.4), and blacklist regions were excluded using BEDTools (v2.29.2). Peaks were called using MACS2 (v2.2.7.1), and quality control metrics, including fragment size distribution and TSS enrichment scores, were assessed using the pipeline's HTML report. The resultant BigWig signal tracks, peak BED files, and BAM files were used for subsequent downstream analysis.

### Mouse and Human Epigenomic Data Analysis to Identify eRNA‐Producing Enhancers with SE Features

Mouse epigenomic data analysis was performed to identify eRNA‐producing enhancers with SE features under inflammatory conditions mimicked by LPS stimulation. First, consensus enhancers were defined by intersecting H3K4me1 and H3K27ac peaks derived from GSE163293 while ensuring these regions did not overlap with promoters. To identify LPS‐specific enhancers, DiffBind (v3.12.0) analysis was conducted using p300 ChIP‐seq signals, generating a list of differentially enriched enhancers based on the following criteria: log_2_FC>0 and adjusted P<0.05. Visualization of these LPS‐specific enhancers was performed using volcano plots with ggplot2 (v3.5.1) and enrichment heatmaps generated by deepTools (v3.5.1).

Next, transcriptionally active enhancers producing eRNAs were identified through TAPE analysis,^[^ ^30^ ^]^ where consensus enhancers served as input peaks, and 1‐h RNA‐seq BAM files generated for this study were utilized. The resulting TAPEs were further analyzed with DiffBind (v3.12.0) using the same criteria (log_2_FC>0 and adjusted P<0.05) to identify LPS‐specific TAPEs. SEs were identified using the ROSE algorithm^[^ ^9^, ^31^ ^]^ based on LPS H3K27ac BAM files and peak files derived from LPS‐stimulated conditions.

Overlap and intersection analysis was performed using the “intersect” function from BEDTools (v2.29.2) to identify LPS‐specific eRNA‐producing enhancers with SE features. Functional annotation of these enhancers was carried out using the ABC model^[^ ^72^ ^]^ to predict enhancer–gene associations. The resulting enhancers were prioritized for downstream functional analyses, including pathway enrichment performed with clusterProfiler (v4.10.1).

Human epigenomic data analysis was conducted using a similar approach to identify eRNA‐producing enhancers with SE features under inflammatory conditions (LPS stimulation). Consensus ATAC‐seq peaks from the GSE100380 dataset were used as input for TAPE analysis,^[^ ^30^ ^]^ along with RNA‐seq BAM files from the GSE172116 dataset. The resulting TAPEs were analyzed using DiffBind (v3.12.0) with RNA‐seq BAM files to identify the LPS‐specific TAPEs based on the following criteria: log_2_FC>0 and adjusted *p* < 0.05. Subsequently, the ROSE algorithm was used to infer the SEs with input peak and BAM files of H3K27ac ChIP‐seq peaks from GSE100380. Overlap analysis, functional annotation, and pathway enrichment for human data were conducted as described for mouse data.

LECIF analysis^[^ ^46^ ^]^ was performed to evaluate the homology between mouse and human LPS‐specific eRNA‐producing enhancers with SE features. To ensure robustness, the resulting enhancers were independently tested using the LECIF score. LECIF score BigWig track files were downloaded from the LECIF GitHub repository (https://github.com/ernstlab/LECIF) for each genome (mm10, hg38). To compare average BigWig signals, GC‐matched random sequences were generated using a custom R script. Visualization of LECIF scores was performed with deepTools (v3.5.1) using the “plotAverage” function. All genomic track displays were generated using the Integrative Genomics Viewer.^[^ ^73^ ^]^

### Blood Collection and Preparation

Blood samples from patients with RA were collected at Gangnam Severance Hospital (Seoul, Korea). The use of these samples was approved by the Institutional Review Board (IRB) of Gangnam Severance Hospital (IRB number: 3‐2024‐0308) in accordance with institutional ethical guidelines. All patients provided written informed consent for the research use of their samples. Blood was collected using BD Vacutainer tubes (BD, 367 856) and centrifuged to separate the serum. The supernatant containing the serum was carefully transferred to a new tube and stored at −80 °C until further analysis. The remaining pellet containing blood cells was resuspended in red blood cell (RBC) lysis buffer (BioLegend, 420 301) and incubated for 15 min at room temperature to lyse the RBCs. After incubation, the samples were centrifuged, and the supernatant was discarded. This process of RBC lysis and centrifugation was repeated until the RBCs were no longer visible in the pellet. And the cell pellet was washed with PBS and centrifuged again under the same conditions.

### PBMCs Isolation

PBMCs were isolated from the whole blood of patients with RA using Ficoll‐Paque density gradient centrifugation. Briefly, blood was diluted with PBS and carefully layered onto Ficoll‐Paque. After centrifugation at 1300 rpm for 20 min without brake, the PBMC layer was collected, washed twice with PBS, and resuspended in Roswell Park Memorial Institute (RPMI)‐1640 medium (Gibco, 11 875 119) supplemented with L‐glutamine (Gibco, 25 030 081) for further experiments.

### RNA Isolation and Real‐Time Quantitative Polymerase Chain Reaction

Total RNA was isolated using TRIzol reagent (Invitrogen, 15 596 018). cDNA was synthesized from 2 µg of RNA using SuperScript IV Reverse Transcriptase (Invitrogen, 18 090 050). qPCR was performed using specific primers, Solg EF‐Taq DNA polymerase (Solgent, SEF16‐R25h), and SYBR Green on the LightCycler 480 system (Roche). GAPDH was used as an endogenous control for normalization. The primer sequences for the target genes are listed in Table S4, Supporting Information.

### Flow Cytometry

For cell surface marker staining, antibodies targeting CD11b (eBioscience, 45‐0112‐82), F4/80 (eBioscience, 25‐4801‐82), Ly6C (eBioscience, 553 104), and Ly6G (eBioscience, 17‐9668‐82) were used. For intracellular Tnfα staining, samples were incubated in conditioned media with ionomycin, phorbol‐12‐myristate‐13‐acetate, and Brefeldin A for 4 h. After incubation, the Fixation/Permeabilization Solution Kit (BD, 554 714) with BD GolgiPlug was used following the manufacturer's instructions. Fixed and permeabilized cells were stained with Tnfα (eBioscience, 12‐7321‐82) antibody. Data was analyzed using FlowJo software.

### Enzyme‐Linked Immunosorbent Assay

To quantify pro‐inflammatory cytokines, samples were collected and centrifuged, and cytokine levels in the supernatants were measured using ELISA MAX Kits from BioLegend for mouse Tnfα (430 204), Il6 (430 504), and human TNFα (430 204).

### Cell Culture

THP‐1 cells, U‐937 and J774A.1 were maintained in RPMI‐1640 medium supplemented with 10% FBS, 1% penicillin (Gibco, 15 070 063), 1 × minimum essential medium‐non‐essential amino acids (Gibco, 11 140 050), 1% L‐glutamine, 1% sodium pyruvate (Gibco, 11 360 070), and 50 µM 2‐mercaptoethanol (Sigma‐Aldrich, M3148).

### ASO Design and Transfection

ASOs used to knock down mouse TNF‐9 eRNA and human DHS44500 were designed and purchased from Qiagen (Antisense LNA GapmeRs). A total of 1 × 10^6^ cells were electroporated with 500 nM ASO using the Neon Transfection System (Thermo Fisher Scientific), following the manufacturer's instructions. The electroporation parameters used were: 1400 V, 20 ms, and 2 pulses for both BMDMs and THP‐1 cells. The cells were immediately transferred to pre‐warmed media without antibiotics. After 48 h, the cells were treated with 100 ng mL^−1^ LPS for 1 h. Following treatment, both cells and cell culture supernatants were collected for qPCR and ELISA analysis, respectively. The mouse TNF‐9 ASO sequences are as follows: negative control ASO: 5′‐AACACGTCTATACGC‐3′; TNF‐9 eRNA ASO1: 5′‐TTAGATTTGAGGTTAC‐3′; TNF‐9 eRNA ASO2: 5′‐GGTTAAACTTGGGTAA‐3′; TNF‐9 eRNA ASO3: 5′‐GGGTGAAGGTTAAACT‐3′; and mouse Tnfα coding region ASO: 5′‐AGGAGCACGTAGTCGG‐3′. The human DHS44500 ASO sequences are as follows: negative control ASO: 5′‐AACACGTCTATACGC‐3′; DHS44500 eRNA ASO1: 5′‐TGATCACTTTAGAGAC‐3′; DHS44500 eRNA ASO2: 5′‐CTTACCCTGTAACTTT‐3′; DHS44500 eRNA ASO3: 5′‐CTTCTTACCCTGTAAC‐3′; and human TNFα coding region ASO: 5′‐ACGTCCCGGATCATGC‐3′.

### Enhancer Silencing via CRISPRi

To investigate the functional relevance of the TNF‐9 (mouse) and DHS44500 (human) enhancer regions, we employed CRISPR interference (CRISPRi) to repress enhancer activity. A lentiviral vector expressing KRAB‐dCas9‐P2A‐mCherry (Addgene #60 954) was kindly provided by Dr. Kyung Hyun Ryu (Division of Biological Sciences, Yonsei University). Guide RNAs targeting the enhancer regions were designed using the CRISPOR tool and cloned into U6 promoter‐driven expression vectors. The following gRNAs were used to target the mouse TNF‐9 enhancer: (1) ATTTGGGTGAAGGTTAAACT (2) TCTAAGCACATACCCCTCAA (3) AGCTCCGGAGCCTGCAAACC The human DHS44500 enhancer was targeted using: (1) CGTGCATGTGAGATATGCGA (2) TCTGCGTGCCTAACACATGC (3) ATTAGCCCTAGAAACAGGGT Electroporation was used to transfect J774A.1 and U937 cells with both the KRAB‐dCas9 construct and corresponding gRNAs. Following puromycin selection, transduced cells were used for downstream analyses.

### Statistical Analysis

All data are presented as mean ± standard error of the mean (SEM), unless otherwise specified. Each experiment was independently repeated at least three times. For normally distributed data, two‐group comparisons were performed using two‐tailed Student's *t*‐test, while comparisons among three or more groups were conducted using one‐way or two‐way ANOVA followed by Tukey's post hoc test. All statistical analyses were performed using GraphPad Prism 8 (version 8.2). Statistical significance is indicated as follows: *p* < 0.05 (), *p* < 0.01 (), *p* < 0.001 (), and *p* < 0.0001 (****).

## Conflict of Interest

The authors declare no conflict of interest.

## Author Contributions

M.C. and S.M.K. contributed equally to this work. M.C. analyzed the data, performed the experiments, and wrote the manuscript. S.M.K. analyzed the data and wrote the manuscript. J.L., W.W., E.L., S.H., and H.J.P. performed the experiments. Y.L., S.H.D., and T.K.K. contributed to the data interpretation. O.C.K. and M.C.P. provided clinical samples. R.A.F. conceived the study and generated KO mouse. L.K.K. conceived the study, analyzed the data and wrote the manuscript. All authors read and approved the final manuscript.

## Supporting information



Supporting Information

Supporting Information

## Data Availability

The sequencing datasets generated in this study have been deposited in the Gene Expression Omnibus (GEO) and are publicly available under the following accession numbers: GSE291297 (LPS 1h RNA‐seq), GSE291298 (LPS 1h histone CUT&RUN), and GSE291300 (HSS9 KO RNA‐seq). Additionally, publicly available datasets were used for analysis. ATAC‐seq datasets include chromatin accessibility data from LPS‐treated human monocytes (GSE100380) and LPS‐stimulated mouse monocyte‐derived macrophages (GSE222804). RNA‐seq datasets include transcriptomic data from LPS‐treated induced pluripotent stem cell‐derived macrophages (GSE172116) and bulk RNA‐seq from mouse bone marrow‐derived macrophages before and after LPS stimulation (GSE163293). Epigenomic datasets from patients with inflammatory diseases were also analyzed, including those from rheumatoid arthritis (CRA002749) and psoriasis (GSE237767 and GSE161076). Further inquiries regarding data availability and access can be directed to the corresponding authors.
