# Supplementary material for: Targeting eRNA‐Producing Super‐Enhancers Regulates TNFα Expression and Mitigates Chronic Inflammation in Mice and Patient‐Derived Immune Cells
Source: Adv Sci (Weinh). 2025 Jul 21;12(39):e05214. doi: 10.1002/advs.202505214 (PMC12533286; doi:10.1002/advs.202505214)
Supplement: Supplementary file 1 — Supporting Information [file ADVS-12-e05214-s001.pdf]

ADVANCED SCIENCE
Open Access

Supporting Information

for *Adv. Sci.*, DOI 10.1002/advs.202505214

Targeting eRNA-Producing Super-Enhancers Regulates TNFα Expression and Mitigates Chronic Inflammation in Mice and Patient-Derived Immune Cells

Minjeong Cho, Su Min Kim, Jiyeon Lee, Oh Chan Kwon, Wonjin Woo, Eunji Lee, Hyo Jin Park, Yeongun Lee, So Hee Dho, Tae-Kyung Kim, Min-Chan Park, Richard A. Flavell and Lark Kyun Kim**

Supporting Information

Targeting eRNA-producing super-enhancers regulates TNFα expression and mitigates chronic inflammation in mice and patient-derived immune cells

Minjeong Cho[1]†, Su Min Kim[1]†, Jiyeon Lee[1], Oh Chan Kwon[2], Wonjin Woo[1], Eunji Lee[1], Hyo Jin Park[1], Yeongun Lee[1], So Hee Dho[1], Tae-Kyung Kim[3], Min-Chan Park[2], Richard A. Flavell[4,5]*, Lark Kyun Kim[1]*

*Corresponding author. Email: LKKIM@yuhs.ac (L.K.K.); richard.flavell@yale.edu (R.A.F.)

Figure S1. Multi-omics characterization of TAPE-associated enhancer activity and transcriptional dynamics upon LPS stimulation.
Fig. S2. Characterization of TNF-9 eRNA transcription and regulatory elements at the Tnfα locus.
Fig. S3. Generation and immune profiling of TNF-9 KO mice at steady state.
Fig. S4. Transcriptomic analysis of TNF-9 KO mouse macrophages reveals differential gene expression and enhancer activity upon LPS stimulation.
Fig. S5. Impact of TNF-9 KO on pro-inflammatory cytokine expression and secretion in BMDMs upon LPS stimulation.
Fig. S6. TNF-9 deletion modulates immune cell activation and cytokine expression in an LPS-induced sepsis model.
Fig. S7. Effect of anti-TNF treatment on immune cell infiltration and skin inflammation in an IMQ-induced psoriasis mouse model.
Figure S8. CRISPR interference at the TNF-9/DHS44500 enhancer attenuates TNFα expression.
Fig. S9. Targeting TNFα-associated eRNA with ASO modulates inflammatory gene expression in THP-1 cells and PBMCs from patients with RA.

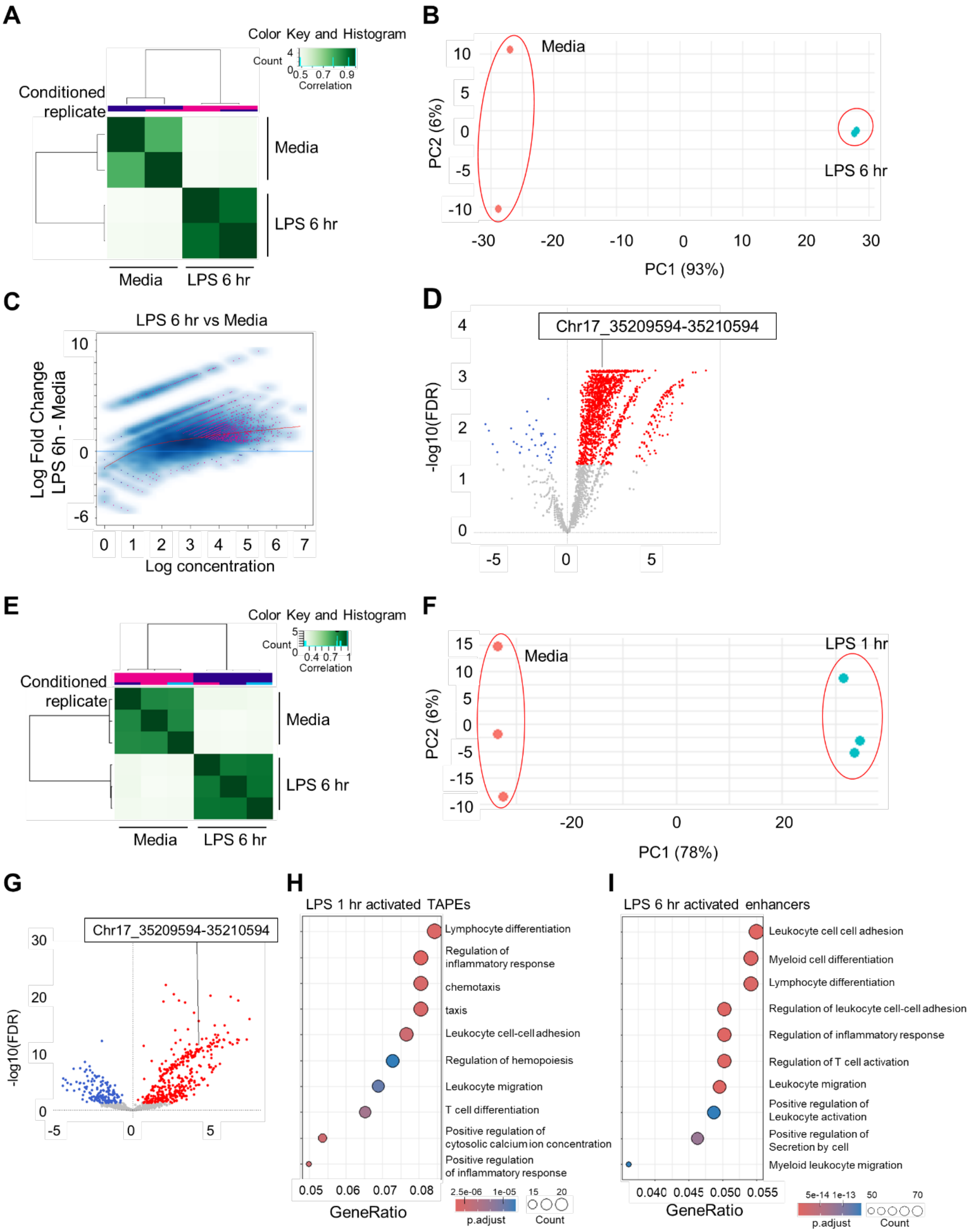
A
Color Key and Histogram
Count
Correlation
Conditioned replicate
Media
LPS 6 hr
B
Media
LPS 6 hr
PC2 (6%)
PC1 (93%)
C
LPS 6 hr vs Media
Log Fold Change LPS 6h - Media
Log concentration
D
Chr17_35209594-35210594
-log10(FDR)
E
Color Key and Histogram
Count
Correlation
Conditioned replicate
Media
LPS 6 hr
F
Media
LPS 1 hr
PC2 (6%)
PC1 (78%)
G
Chr17_35209594-35210594
-log10(FDR)
H
LPS 1 hr activated TAPEs
Lymphocyte differentiation
Regulation of inflammatory response
chemotaxis
taxis
Leukocyte cell-cell adhesion
Regulation of hemopoiesis
Leukocyte migration
T cell differentiation
Positive regulation of cytosolic calcium ion concentration
Positive regulation of inflammatory response
GeneRatio
p.adjust
Count
I
LPS 6 hr activated enhancers
Leukocyte cell cell adhesion
Myeloid cell differentiation
Lymphocyte differentiation
Regulation of leukocyte cell-cell adhesion
Regulation of inflammatory response
Regulation of T cell activation
Leukocyte migration
Positive regulation of Leukocyte activation
Positive regulation of Secretion by cell
Myeloid leukocyte migration
GeneRatio
p.adjust
Count

Figure S1. Multi-omics characterization of TAPE-associated enhancer activity and transcriptional dynamics upon LPS stimulation.

(**A**) Sample distance heatmap and (**B**) PCA plot of publicly available p300 ChIP-seq datasets, constructed using consensus TAPE regions. (**C**) MA and (**D**) volcano plots illustrating differential p300 ChIP-seq signals. In (**D**), significantly upregulated TAPE regions (false discovery rate [FDR] < 0.05) are highlighted in red, whereas significantly downregulated regions are in blue. (**E**) Sample distance heatmap and (**F**) PCA plot of bulk RNA-seq datasets based on the consensus TAPE regions. (**G**) Volcano plot displaying differentially expressed eRNAs mapped to TAPE regions, with significantly upregulated eRNAs (FDR < 0.05) in red and significantly downregulated eRNAs in blue. (**H**) Pathway enrichment analysis of GOBP terms for genes proximal to TAPEs activated 1 hr after LPS stimulation. (**I**) Pathway enrichment analysis of genes proximal to TAPEs activated 6 hr after LPS stimulation.

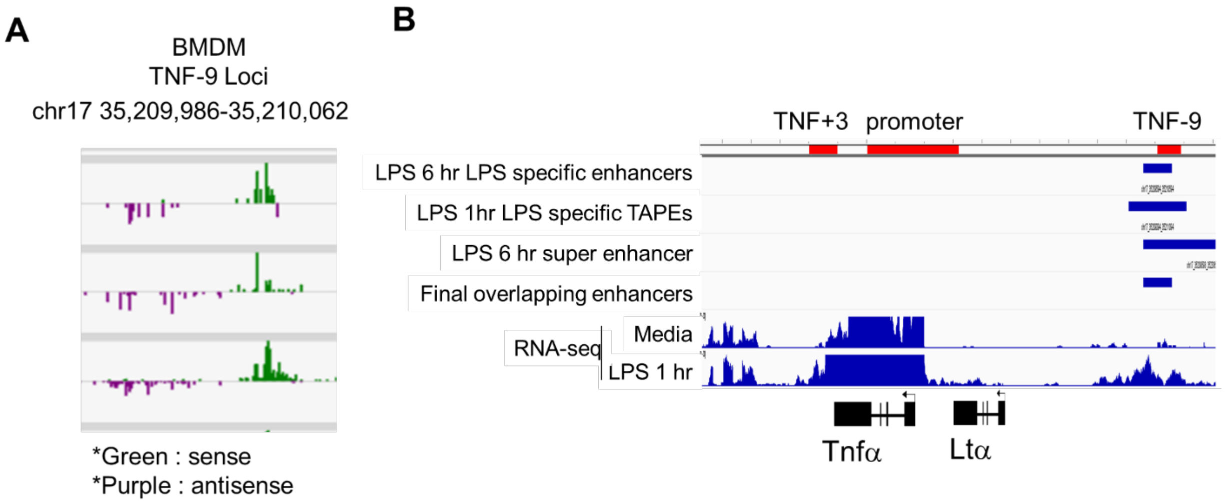

Figure S2. Characterization of TNF-9 eRNA transcription and regulatory elements at the Tnfα locus.

(**A**) CAGE-seq data demonstrating that TNF-9 eRNA is a bi-directional transcript. (**B**) UCSC Genome Browser view of the *Tnfα* and *Ltα* loci, highlighting the HSS+3, HSS-0.8, and HSS-9 regions within the mm10 mouse genome assembly.

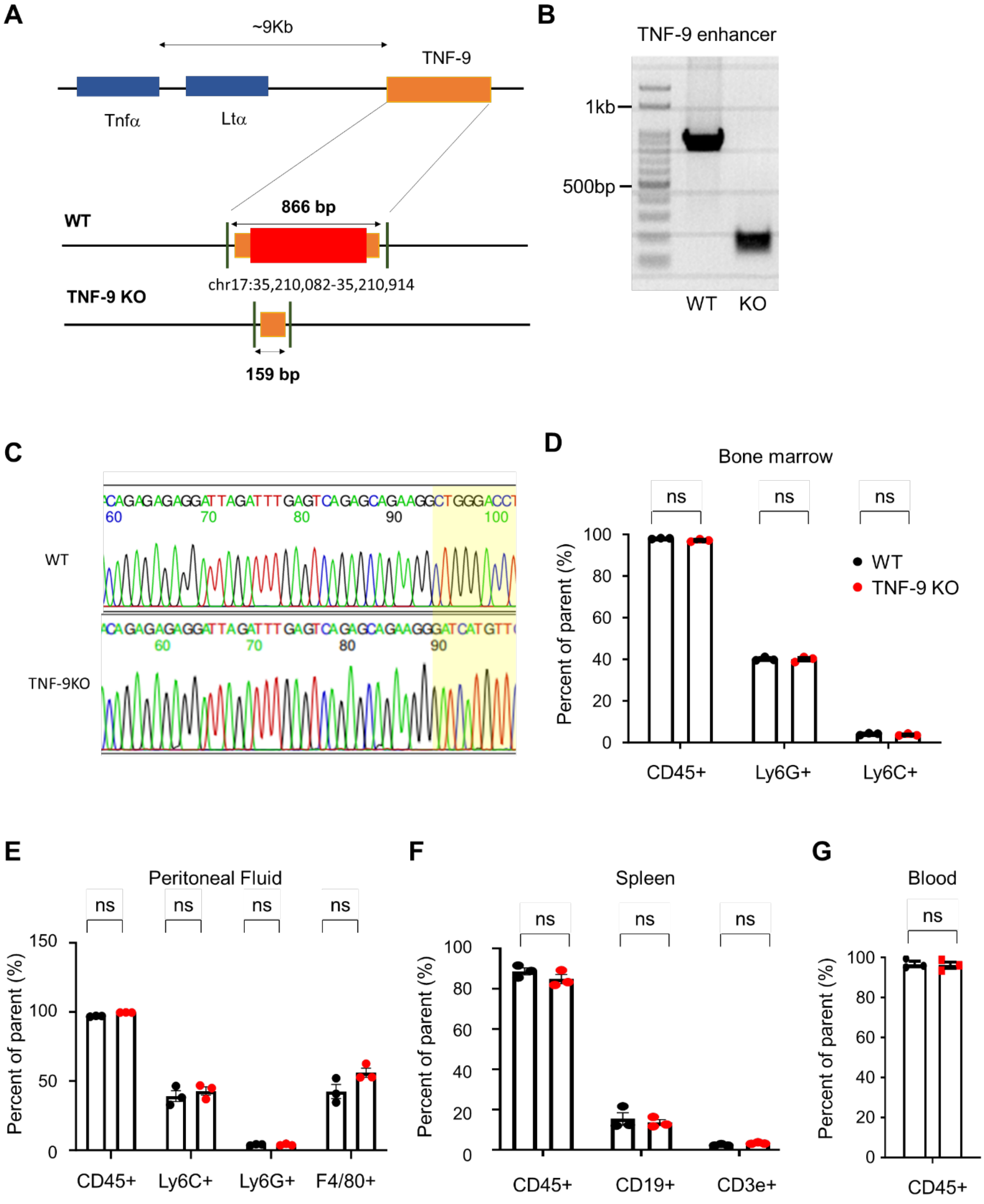
A
~9Kb
TNF-9
Tnfα
Ltα
WT
866 bp
chr17:35,210,082-35,210,914
TNF-9 KO
159 bp
B
TNF-9 enhancer
1kb
500bp
WT
KO
C
WT
TNF-9KO
D
Bone marrow
ns
Percent of parent (%)
WT
TNF-9 KO
CD45+
Ly6G+
Ly6C+
E
Peritoneal Fluid
CD45+
Ly6C+
Ly6G+
F4/80+
F
Spleen
CD45+
CD19+
CD3e+
G
Blood
CD45+

Figure S3. Generation and immune profiling of TNF-9 KO mice at steady state.

(**A**) Strategy for generating TNF-9 KO mice. The TNF-9 locus is located 9 kb upstream of the *Tnfα* gene. (**B**) Genotyping results confirming an 866 bp deletion in KO mice. (**C**) Representative Sanger sequencing chromatograms validating the deletion of the TNF-9 region. The highlighted yellow region indicates the junction site where the deletion begins in the KO allele. (**D**)Percentage of $CD45^+$, $Ly6G^+$, and $Ly6C^+$ cells in BM at steady state. (**E**) Percentage of $CD45^+$, $Ly6C^+$, $Ly6G^+$, and $F4/80^+$ cells in peritoneal fluid at steady state. (**F**) Percentage of $CD45^+$, $CD19^+$, and $CD3e^+$cells in spleen at steady state. (**G**) Percentage of $CD45^+$ cells in blood at steady state. Data were collected from 7-week-old male mice. Data are presented as mean ± SEM. Statistical significance was determined using Student's *t*-test.

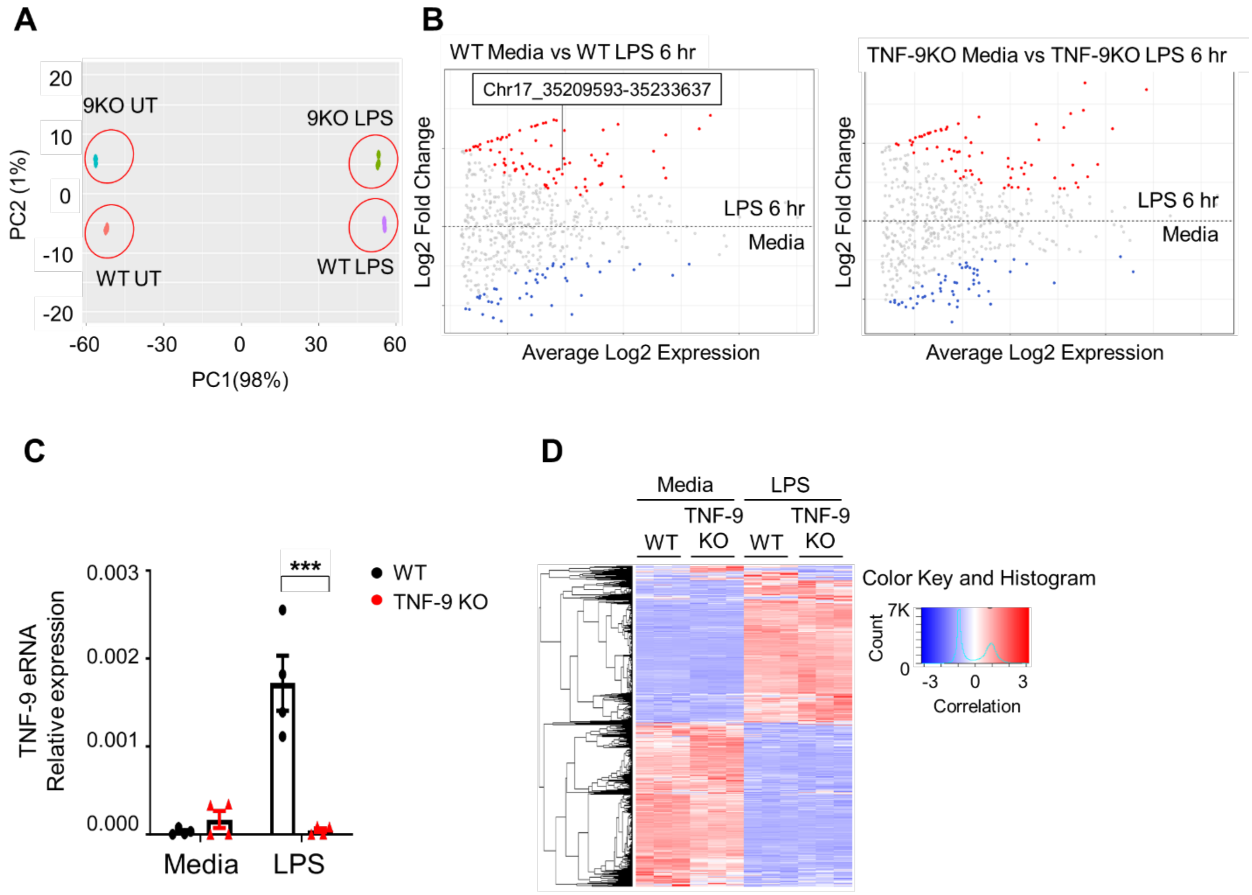

Figure S4. Transcriptomic analysis of TNF-9 KO mouse macrophages reveals differential gene expression and enhancer activity upon LPS stimulation.

(**A**) PCA plot showing the clustering of TNF-9 KO and WT cells under UT and LPS-treated conditions. (**B**) MA plots showing differential expression between TNF-9 KO and WT cells treated with LPS for 6 h, based on RNA-seq BAM files aligned to TAPEs. Significantly expressed regions (highest fold change after LPS treatment) are highlighted in red and blue, with the TNF-9 enhancer region (chr17:35209593-35233637) indicated by a line. (**C**) Expression levels of TNF-9 eRNA in BMDMs from WT and TNF-9 KO mice following LPS treatment for 6 h. Data are presented as mean ± SEM. Statistical significance was determined using Student's *t*-test. (***P<0.001). (**D**) Heatmap showing hierarchical clustering of gene expression profiles from TNF-9 KO and WT cells under UT and LPS-treated conditions.

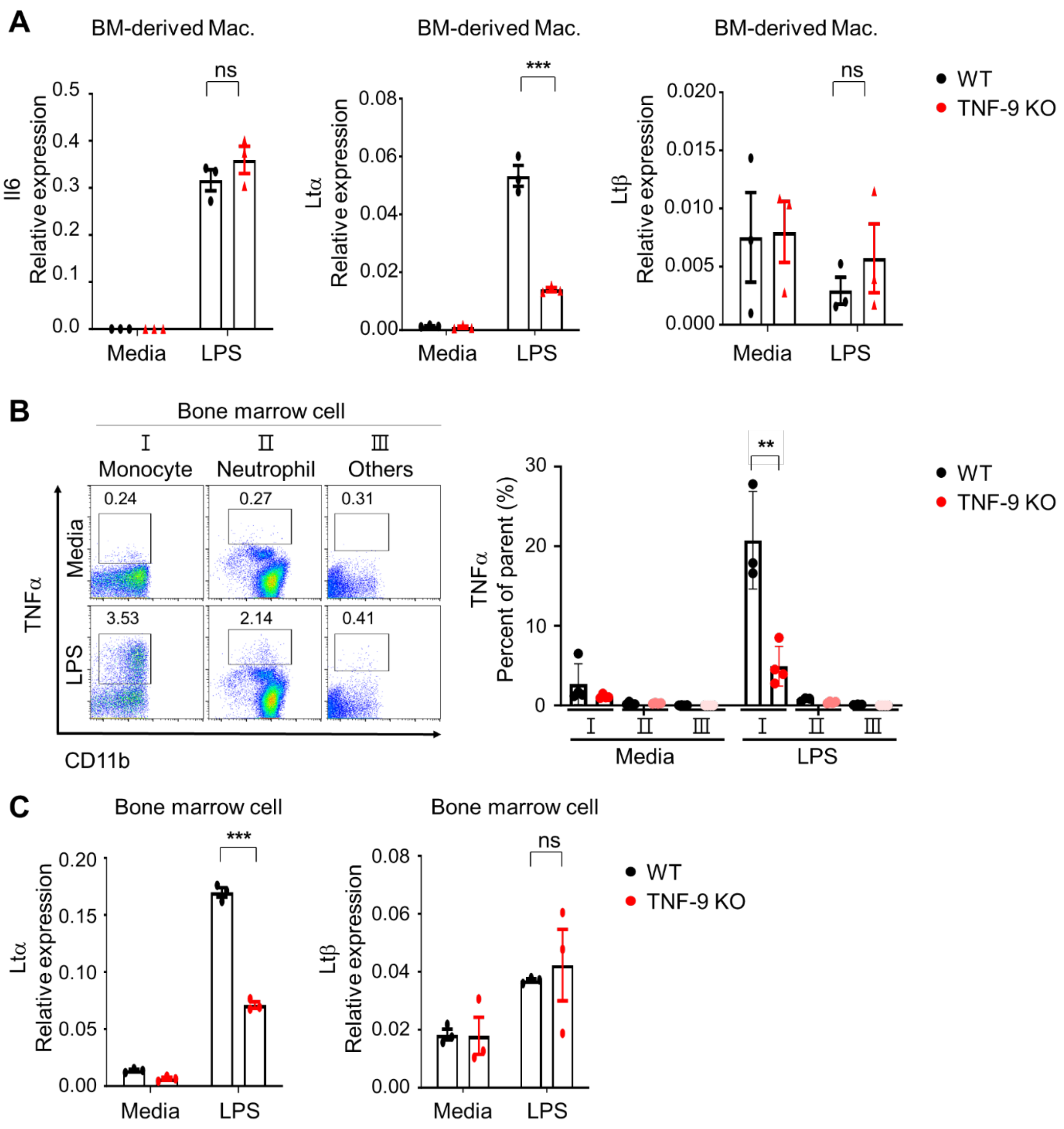

Figure S5. Impact of TNF-9 KO on pro-inflammatory cytokine expression and secretion in BMDMs upon LPS stimulation.

(**A**) Expression levels of *Il6*, *Ltα*, and *Ltβ* in BMDMs following LPS treatment. (**B**) TNFα-expressing population in WT BMs following LPS treatment. The percentage of CD11b$^+$ TNFα$^+$ cells in WT and TNF-9 KO mice is shown. (**C**) Expression levels of *Ltα* and *Ltβ* in BMs following LPS treatment, assessed by quantitative polymerase chain reaction. Data are presented as mean ± SEM. Statistical significance was determined using Student's *t*-test. (*P<0.05, **P<0.01, and ***P<0.001).

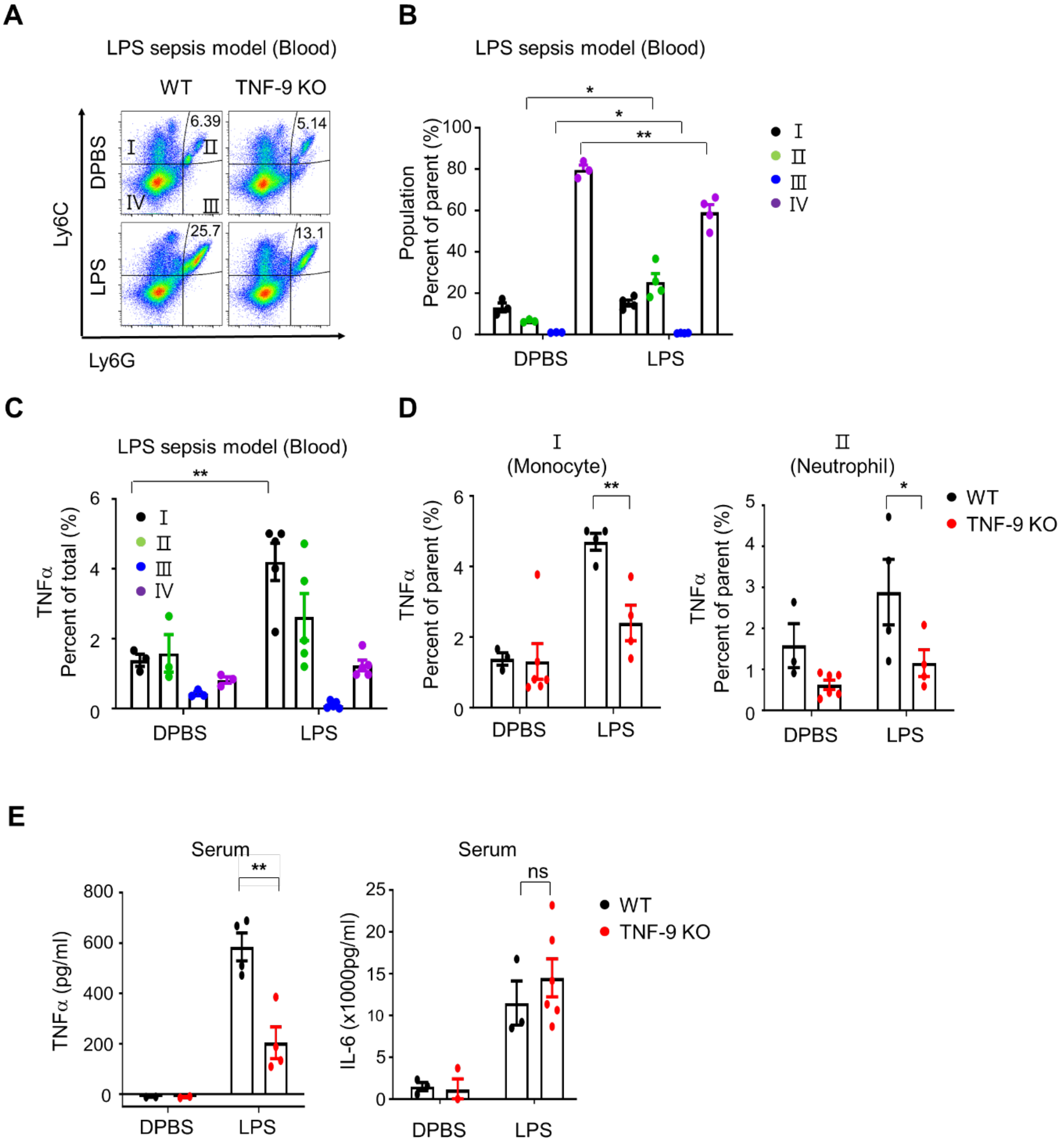

Figure S6. TNF-9 deletion modulates immune cell activation and cytokine expression in an LPS-induced sepsis model.

(**A** and **B**) Activated cell populations in the blood of WT and TNF-9 KO mice in the LPS-induced sepsis model, identified by flow cytometry, following intravascular LPS injection. Cells were gated to identify specific populations, including Ly6C$^+$ monocytes (Population I) and Ly6C$^+$Ly6G$^+$ neutrophils (Population II). (**C**) Percentage of TNFα$^+$ cells analyzed within each population in WT mice. (**D**) Comparison of Tnfα expression in Ly6C$^+$ monocytes and Ly6C$^+$Ly6G$^+$ neutrophils between WT and TNF-9 KO mice. (**E**) Serum cytokine levels of Tnfα

and Il6. Data are presented as mean ± SEM. Statistical significance was determined using Student's *t*-test. (*P<0.05 and **P<0.01).

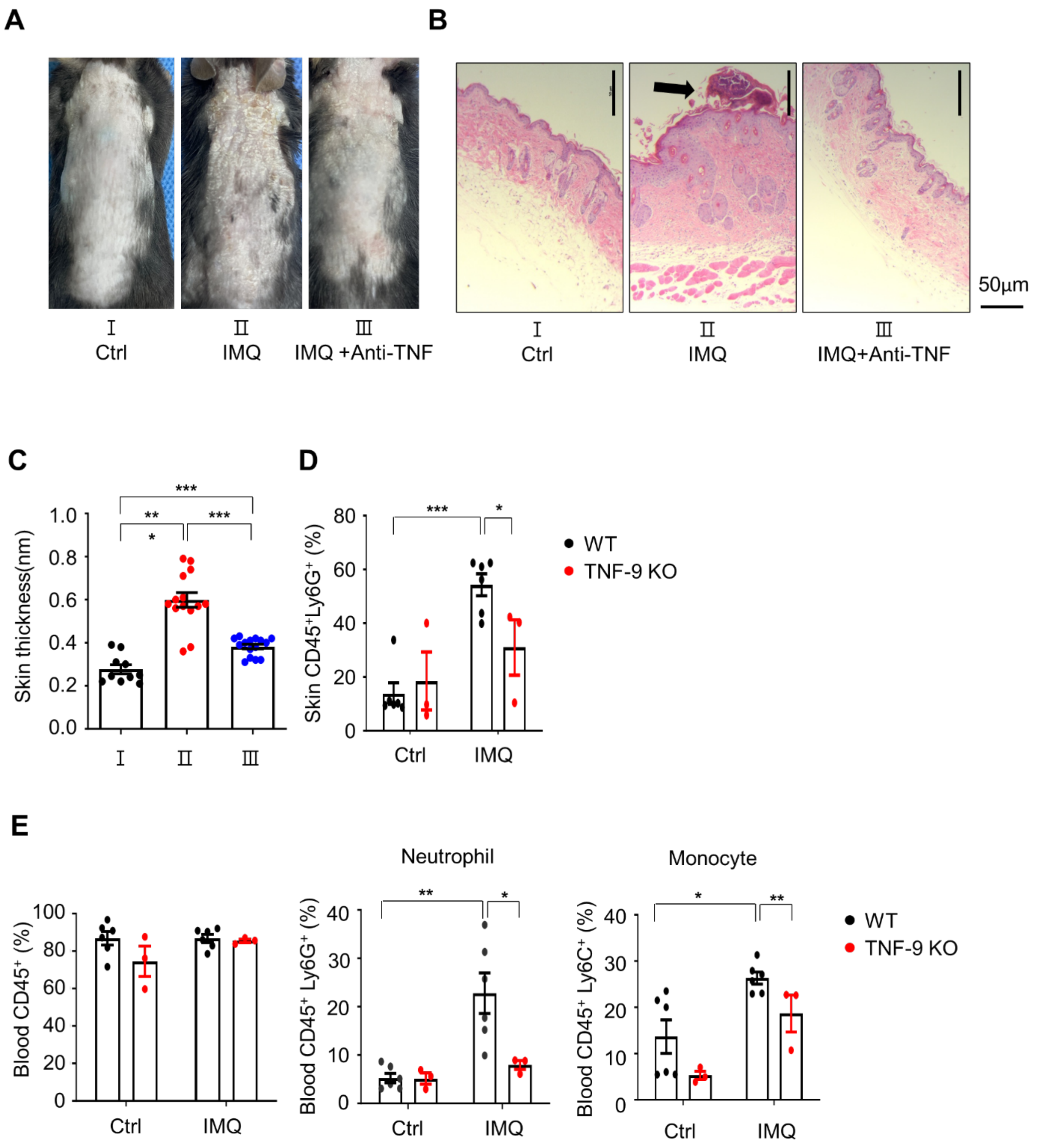

Figure S7. Effect of anti-TNF treatment on immune cell infiltration and skin inflammation in an IMQ-induced psoriasis mouse model.

(**A**) Symptom alleviation in the IMQ-induced psoriasis mouse model following anti-TNF treatment. (**B**) Hematoxylin and eosin staining of skin sections. Scale bar: 50 µm. (**C**) Quantification of skin thickness. (**D**) Flow cytometric analysis of CD45$^+$ Ly6G$^+$ populations in skin. (**E**) Flow cytometric analysis of CD45$^+$, CD45$^+$ Ly6G$^+$ neutrophils, and CD45$^+$ Ly6C$^+$ monocytes in blood. Data are

presented as mean ± SEM. Statistical significance was determined using Student's *t*-test. (*P<0.05, **P<0.01, and ***P<0.001).

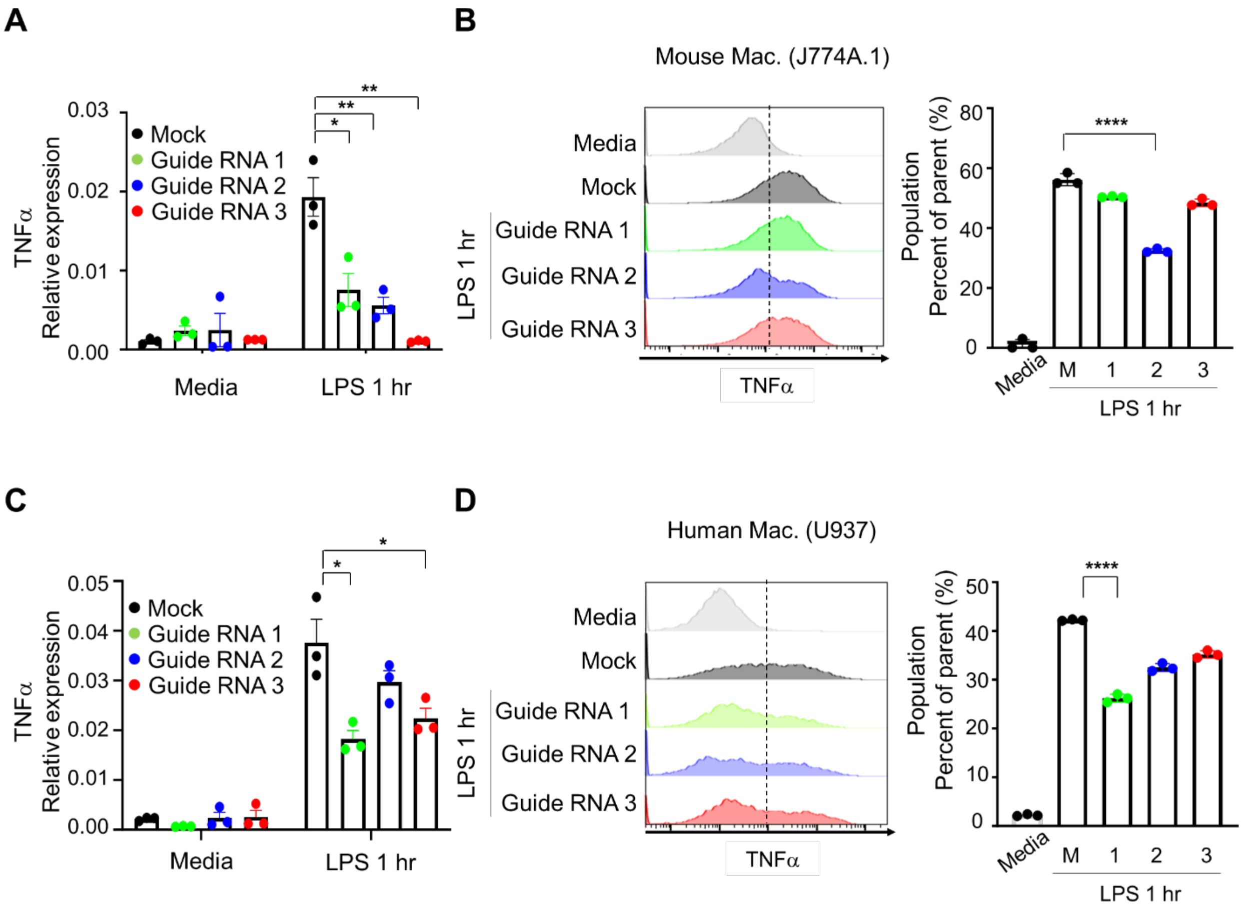

Figure S8. CRISPR interference at the TNF-9/DHS44500 enhancer attenuates TNFα expression.

(A–B) J774A.1 cells were electroporated with three sgRNAs targeting the TNF-9 enhancer region and subsequently stimulated with LPS. TNFα expression was assessed at both the mRNA level by qPCR and the protein level by intracellular FACS analysis. (C–D) U937 cells were electroporated with three sgRNAs targeting the DHS44500 enhancer region and subsequently stimulated with LPS. TNFα expression was assessed at both the mRNA level by qPCR and the protein level by intracellular FACS analysis.

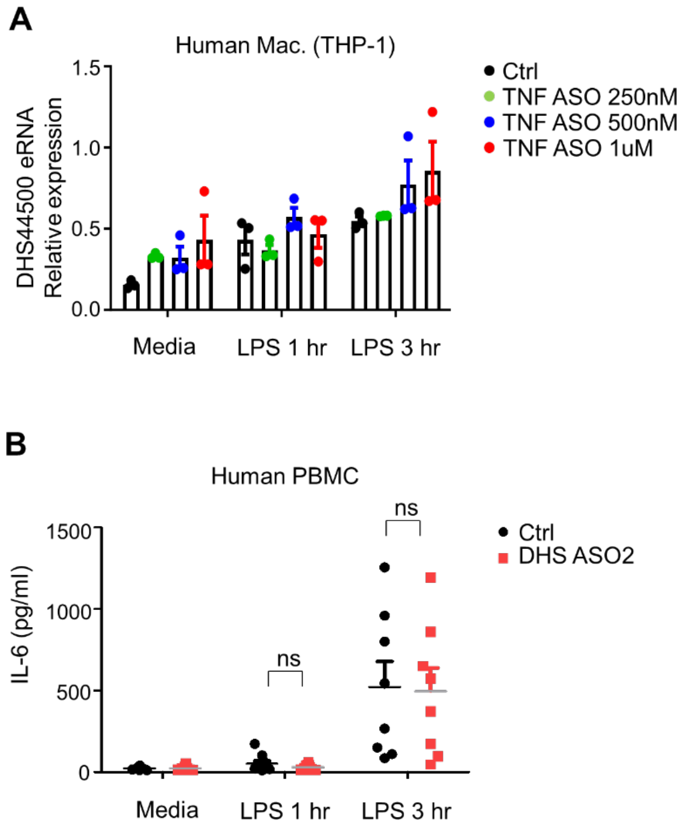

Figure S9. Targeting TNFα-associated eRNA with ASO modulates inflammatory gene expression in THP-1 cells and PBMCs from patients with RA.

(A) THP-1 cells were electroporated with three candidate ASOs targeting the TNFα coding region, followed by LPS stimulation to induce cytokine expression. The expression level of DHS44500 eRNA was analyzed after ASO treatment. (B) Protein levels of IL-6 were evaluated following DHS44500 ASO2 treatment of PBMCs from patients with RA. (n=10); Student's *t*-test.
