# Supplementary material for: Targeting eRNA‐Producing Super‐Enhancers Regulates TNFα Expression and Mitigates Chronic Inflammation in Mice and Patient‐Derived Immune Cells
Source: Adv Sci (Weinh). 2025 Jul 21;12(39):e05214. doi: 10.1002/advs.202505214 (PMC12533286; doi:10.1002/advs.202505214)
Supplement: Supplementary file 2 — Supporting Information [file ADVS-12-e05214-s002.docx]

**Table S1.** List of target genes associated with SEs in mouse upon LPS treatment

| Rank | | Enhancer | | Target gene | | ABC score | |
| --- | --- | --- | --- | --- | --- | --- | --- |
| 1 | chr17 | | 88131930 | 88132931 | Gm4832 | | 0.294643 |
| 2 | chr2 | | 167925426 | 167926427 | Ptpn1 | | 0.277923 |
| 3 | chr17 | | 35209593 | 35210594 | Ltα | | 0.237012 |
| 4 | chr3 | | 95650530 | 95651531 | Mcl1 | | 0.222416 |
| 5 | chr2 | | 167357675 | 167358676 | B4galt5 | | 0.211222 |
| 6 | chr17 | | 35209593 | 35210594 | Mir6974 | | 0.210106 |
| 7 | chr1 | | 39578589 | 39579590 | Rnf149 | | 0.207645 |
| 8 | chr15 | | 103369702 | 103370703 | Itga5 | | 0.171966 |
| 9 | chr17 | | 35209593 | 35210594 | Tnfα | | 0.154464 |
| 10 | chr5 | | 90897716 | 90898717 | Cxcl2 | | 0.15136 |
| 11 | chr5 | | 90897716 | 90898717 | Cxcl1 | | 0.146224 |
| 12 | chr17 | | 88123601 | 88124602 | Gm4832 | | 0.138744 |
| 13 | chr3 | | 95650530 | 95651531 | Ensa | | 0.129346 |
| 14 | chr4 | | 59253780 | 59254781 | Gm12596 | | 0.125342 |
| 15 | chr17 | | 17866443 | 17867444 | Has1 | | 0.123371 |
| 16 | chr9 | | 65541570 | 65542571 | Ankdd1a | | 0.120543 |
| 17 | chr9 | | 65535457 | 65536458 | Ankdd1a | | 0.118496 |
| 18 | chr1 | | 39578589 | 39579590 | Rnf149 | | 0.116076 |
| 19 | chr12 | | 111437426 | 111438427 | Tnfaip2 | | 0.113124 |
| 20 | chr13 | | 43774594 | 43775595 | Cd83 | | 0.111684 |

**Table S2.** Gene list showing differential expression between WT and TNF-9 KO under LPS treatment conditions

| rank | symbol | baseMean | log2FoldChange | pvalue | padj |
| --- | --- | --- | --- | --- | --- |
| 1 | Mid1 | 516.4845 | -3.99295 | 9.95030573242689e-127 | 1.22597716929232e-122 |
| 2 | Pira2 | 315.9246 | -4.08046 | 1.55727596243423e-69 | 9.59359856657609e-66 |
| 3 | Ifi208 | 833.8746 | -7.94731 | 3.91187950148563e-65 | 1.60660891126015e-61 |
| 4 | Tnfα | 10824.75 | -1.07352 | 6.38124005508695e-63 | 1.96558146796816e-59 |
| 5 | Fcrl2 | 1960.406 | 1.228428 | 1.23247159408979e-61 | 3.03705650215605e-58 |
| 6 | Adam19 | 252.9846 | 2.451404 | 3.20770234444356e-55 | 6.58701676431484e-52 |
| 7 | Rgs1 | 4379.683 | -1.10528 | 5.33928971432329e-53 | 9.39791265288247e-50 |
| 8 | Apoe | 73346.91 | 1.195628 | 1.00623894216089e-45 | 1.54973375079554e-42 |
| 9 | Tmem176b | 2370.184 | 1.026961 | 1.09343436481461e-37 | 1.4969116454312e-34 |
| 10 | C1qa | 9972.675 | 1.091971 | 2.12097267590427e-37 | 2.61325043398165e-34 |
| 11 | Gdf3 | 200.662 | -1.82347 | 4.74099510036665e-26 | 3.65086253947609e-23 |
| 12 | Slc15a2 | 105.1624 | -3.4695 | 5.34878433837648e-26 | 3.87661010783156e-23 |
| 13 | Acta2 | 181.9109 | 1.766588 | 1.49104293185301e-23 | 8.35051816516408e-21 |
| 14 | Pira11 | 319.128 | -1.56803 | 4.90133560784029e-22 | 2.51622316767501e-19 |
| 15 | H4c18 | 846.5049 | -1.27599 | 5.45648202218644e-21 | 2.58574288443689e-18 |
| 16 | Abca9 | 836.2209 | 1.008186 | 9.45782339956273e-18 | 3.53120733654583e-15 |
| 17 | Camk2b | 69.02081 | 3.645551 | 2.48284841019198e-17 | 8.99740448881631e-15 |
| 18 | Dio2 | 378.7893 | -1.65344 | 3.99043800467086e-15 | 1.11741333308067e-12 |
| 19 | Ccn2 | 147.3574 | 1.438604 | 1.23196536599744e-13 | 2.52984087907574e-11 |
| 20 | Lrmda | 233.7581 | -1.36397 | 1.71777543095534e-13 | 3.25610939766165e-11 |

**Table S3.** List of target genes associated with SEs in human upon LPS treatment

| Rank | | Enhancer | | Target gene | | ABC score | |
| --- | --- | --- | --- | --- | --- | --- | --- |
| 1 | chr15 | | 79976805 | 79979945 | BCL2A1 | | 0.611059 |
| 2 | chr2 | | 1.43E+08 | 1.43E+08 | KYNU | | 0.581859 |
| 3 | chr1 | | 2.44E+08 | 2.44E+08 | C1orf100 | | 0.37569 |
| 4 | chr18 | | 48951992 | 48953493 | SMAD7 | | 0.349243 |
| 5 | chr17 | | 34251867 | 34253368 | CCL2 | | 0.326476 |
| 6 | chr4 | | 38785534 | 38787035 | TLR10 | | 0.301066 |
| 7 | chrX | | 43975653 | 43977154 | NDP | | 0.282627 |
| 8 | chr10 | | 23432034 | 23434693 | OTUD1 | | 0.273331 |
| 9 | chr9 | | 1.21E+08 | 1.21E+08 | TRAF1 | | 0.260137 |
| 10 | chr16 | | 70803976 | 70805477 | VAC14 | | 0.250928 |
| 11 | chr6 | | 14108368 | 14109869 | CD83 | | 0.228226 |
| 12 | chr17 | | 75294542 | 75296641 | SLC25A19 | | 0.217062 |
| 13 | chr1 | | 1.53E+08 | 1.53E+08 | S100A8 | | 0.216269 |
| 14 | chr10 | | 61896410 | 61899973 | ARID5B | | 0.212999 |
| 15 | chr11 | | 59216262 | 59217763 | MPEG1 | | 0.206485 |
| 16 | chr6 | | 46770428 | 46774368 | MEP1A | | 0.206324 |
| 17 | chr19 | | 39401944 | 39404696 | ZFP36 | | 0.204829 |
| 18 | chr7 | | 36736164 | 36737665 | AOAH | | 0.19708 |
| 19 | chr1 | | 2.03E+08 | 2.03E+08 | LINC01353 | | 0.195864 |
| 20 | chr19 | | 14784614 | 14786115 | ADGRE2 | | 0.192872 |

*** SEs expressing eRNAs in LPS-activated human monocytes were identified and their nearest target genes were determined. SE chromosomal positions (chromosome, start, and end) and the target gene's chromosomal positions (chromosome, TSS) are shown. Only SEs with a TSS within 10 kb were included. For genes associated with multiple SEs, only the highest-ranking SE is shown, and the total number of associated SEs is indicated.**

**Table S4.** List of the qPCR primer sequences

| Gene name | Forward sequence | Reverse sequence |
| --- | --- | --- |
| Gapdh | TCGCTCCTGGAAGATGGTGATGG | GGCAAATTCAACGGCACAGTCAAG |
| Tnfα | ATGTCCATTCCTGAGTTCTC | AATCTGGAAAGGTCTGAAGG |
| Ltα | CTCTCTGGTGTCCGCTTCTC | CTGGGGTACCCAACAAGGTG |
| Ltβ | ACGGGTCGTTATGGTACACG | CCCTCTCCTGTAGTCCACCA |
| Il6 | CCAAGACCATCCAATTCATC | CCACAAACTGATATGCTTAGG |
| TNF-9 eRNA | GGAGTCTTTATGCTTCCTGTTGC | AATACAGCTTAGCCAGCGTCT |
| GAPDH | AATCCCATCACCATCTTCCA | TGGACTCCACGACGTACTCA |
| TNFα | CACCACTTCGAAACCTGGGA | TGTAGGCCCCAGTGAGTTCT |
| LTα | TCTTTGGAGCCTTCGCTCTG | GGCAGAATGGGGAGAAGGTC |
| DHS44500 eRNA | ACCCAGGGAAAGTCCCAAAC | TGGGCCAATCTTGGTTTCAAT |
